# Polyphenols targeting multiple molecular targets and pathways for the treatment of vitiligo

**DOI:** 10.3389/fimmu.2024.1387329

**Published:** 2024-07-25

**Authors:** Yixuan Yang, Yanyuan Du, Bingnan Cui

**Affiliations:** Department of Dermatology, Guang’anmen Hospital, China Academy of Chinese Medical Sciences, Beijing, China

**Keywords:** vitiligo, polyphenols, mechanisms, oxidative stress, immunity

## Abstract

Vitiligo, a pigmentary autoimmune disorder, is marked by the selective loss of melanocytes in the skin, leading to the appearance of depigmented patches. The principal pathological mechanism is the melanocyte destruction mediated by CD8^+^ T cells, modulated by oxidative stress and immune dysregulation. Vitiligo affects both physical health and psychological well-being, diminishing the quality of life. Polyphenols, naturally occurring compounds with diverse pharmacological properties, including antioxidant and anti-inflammatory activities, have demonstrated efficacy in managing various dermatological conditions through multiple pathways. This review provides a comprehensive analysis of vitiligo and the therapeutic potential of natural polyphenolic compounds. We examine the roles of various polyphenols in vitiligo management through antioxidant and immunomodulatory effects, melanogenesis promotion, and apoptosis reduction. The review underscores the need for further investigation into the precise molecular mechanisms of these compounds in vitiligo treatment and the exploration of their combination with current therapies to augment therapeutic outcomes.

## 1 Introduction

**Figure 1:**
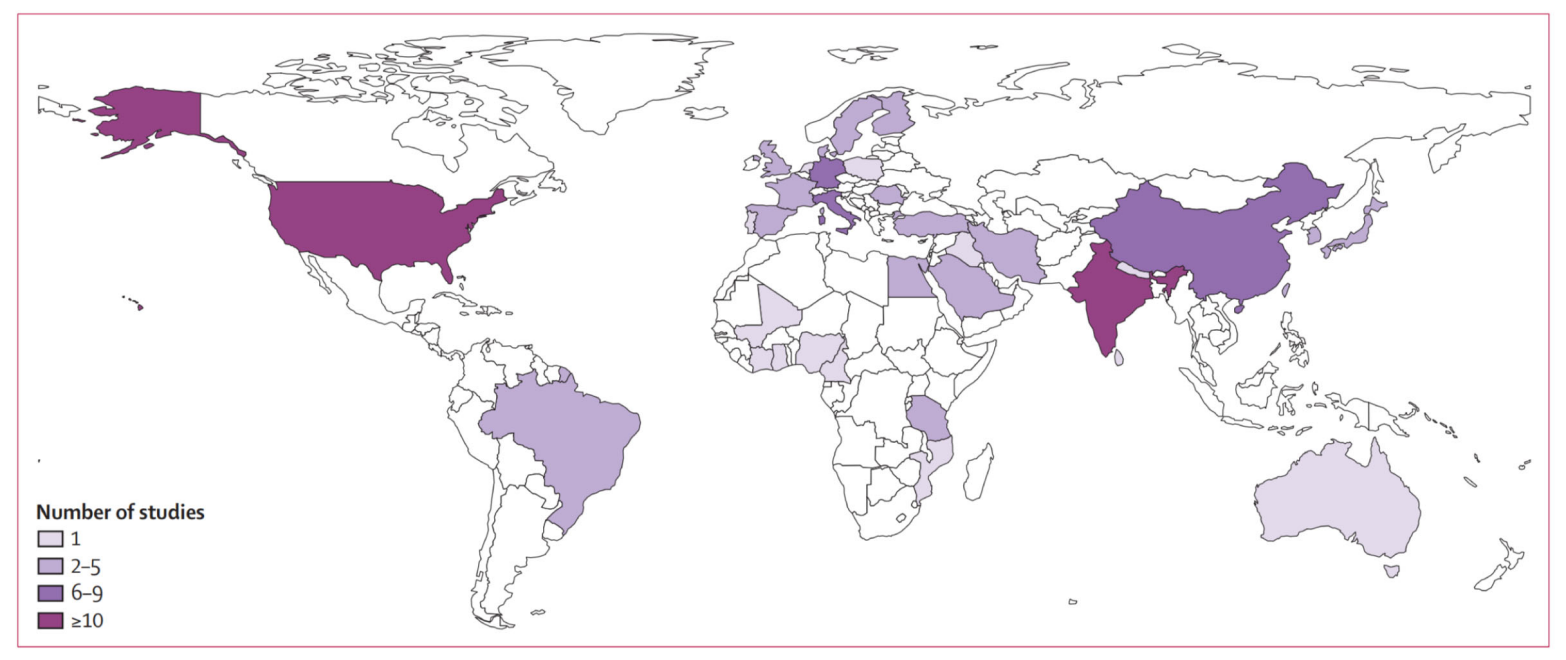
Distribution of studies included in the analysis of vitiligo prevalence. Reprinted with permission from ref (7). Copyright © 2024 The Author(s). Published by Elsevier Ltd.

Vitiligo, an autoimmune disorder, is characterized by the selective loss of melanocytes, leading to depigmentation of skin, which appears as white patches (1). Globally, approximately 0.5–2% of the population is affected by vitiligo, with its prevalence exhibiting no significant variation across genders, races, or geographic regions (2–6). The results of distribution of studies included in the analysis of vitiligo prevalence are depicted in **Figure 1** (7). This condition not only impacts the physical health of patients but also significantly affects their mental well-being, resulting in a diminished quality of life (8). Hence, vitiligo should not be perceived solely as a cosmetic or trivial concern, but rather as a condition capable of causing considerable psychological distress, imposing a substantial burden on the patient (9). Enhanced understanding of the pathogenesis of vitiligo which may facilitate the development of more effective therapeutic strategies, could bring positive benefits to individuals suffering from vitiligo.

**Figure 2:**
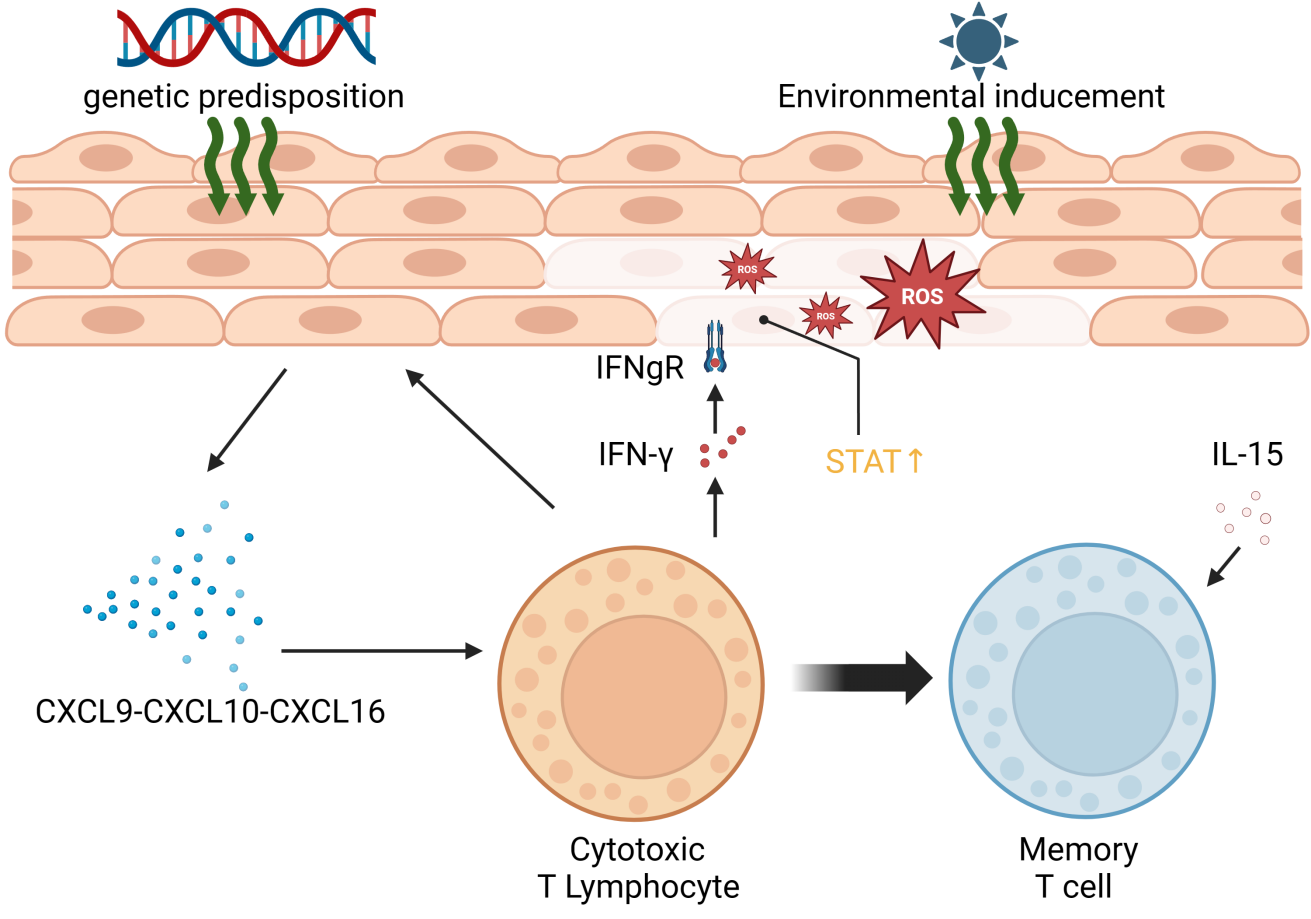
Summary of the pathogenesis of vitiligo.

Vitiligo primarily affects melanocytes, the pigment-producing cells located in the epidermis between hair follicles. Autoimmunity is a key factor in its development. Vitiligo results from the progressive autoimmune destruction of cutaneous melanocytes by CD8^+^ T cells (10). The presence of numerous melanocyte-reactive cytotoxic T cells in the peripheral blood of vitiligo patients, along with perifocal T-cell infiltration causing melanocyte loss, is closely associated with vitiligo development. This process is further enhanced by the secretion of IFN-γ by T cells, stimulating the production of CXCL9 and CXCL10 by keratinocytes and the recruitment of more T cells (11). IFN-γ signaling can activate downstream IFN-γ-inducible genes by binding to the cell surface receptor, forming a complex of two different proteins that recruits JAK1 and JAK2 kinases, resulting in STAT phosphorylation and nuclear translocation (12, 13). Vitiligo often recurs, possibly due to the activity of CD8 tissue-resident memory T cells (TRM), which are responsible for its reactivation. TRM can detect self-antigens in the skin during the stable phase of the disease, leading to the production of IFN-γ, CXCL9, CXCL10, and TNF-α (14). Additionally, interleukin-15 plays a role in maintaining TRM (15). Oxidative stress is also a significant factor in vitiligo. When melanocytes experience stress, they release reactive oxygen species (ROS), leading to an imbalance of pro-oxidants (superoxide dismutase, malondialdehyde, xanthine oxidase) and enzymatic and non-enzymatic antioxidants (catalase, glutathione reductase, glutathione peroxidase, thioredoxin reductase, thioredoxin, superoxide dismutase, as well as the restorative enzymes methionine sulfoxide reductase A and B). This process accelerates the cellular pre-senescent state (16, 17). Furthermore, the production and accumulation of ROS induce DNA damage, protein oxidation and fragmentation, as well as lipid peroxidation, all of which impair cellular function (18). The mechanisms underlying vitiligo are depicted in **Figure 2**.

Current management of vitiligo mainly includes drug therapy, phototherapy, surgical treatment, and self-management of patients (19, 20). These treatments aim to halt the progression of pigment loss, restore skin color, and improve the psychological well-being of patients. However, the existing treatment options have some limitations. 1) Drug therapy: Commonly used drugs include corticosteroids and immunomodulators. Although these drugs can control the condition to some extent, long-term use may lead to a series of side effects, such as skin atrophy, immunosuppression, and the risk of rebound depigmentation if treatment is suddenly stopped. In addition, the effect of drug therapy varies from person to person, and some patients may not achieve the desired treatment outcomes. 2) Phototherapy: Phototherapy such as NB-UVB can promote repigmentation. However, it requires regular treatments over a long period of time and carries risks of sunburn and skin cancer. Excimer laser may be effective for localized areas but could be costly and impractical for larger areas. 3) Surgical treatment: Although skin grafting may be effective for certain patients with localized and stable vitiligo, its application scope is limited by issues such as surgical risks, the survival of transplanted skin, and high costs. 4) Depigmentation therapy: When vitiligo covers a large area, it may be considered to depigment the remaining normal skin to create a uniform skin tone. However, this is a radical measure that is irreversible. 5) Self-management: Although self-management of patients is important, it is often challenging to sustain long term. For example, maintaining good lifestyle habits, avoiding excessive exposure to the sun, and fostering a positive mindset all influence the condition of vitiligo. However, in practice, patients may struggle to adhere to these practices due to various reasons. Therefore, while progress has been made in the management of vitiligo, there are still numerous limitations, including limited efficacy, side effects and risks, economic burden, and a lack of personalized treatment options.

Polyphenols, widely distributed in nature, are increasingly recognized for their pivotal role in safeguarding human health. They derive their name from the multiple phenolic hydroxyl groups present in their structures. Polyphenols can be broadly categorized into phenolic acids, flavonoids, tannins, stilbenes, and lignans based on their chemical composition. These compounds display a broad spectrum of pharmacological effects, encompassing antioxidant, anti-inflammatory, anti-tumor, anti-proliferative, and anti-angiogenic properties (21–25). Various polyphenols have also shown effectiveness in treating diverse types of skin diseases through multiple mechanisms (25, 26).

Compared with other natural compounds, polyphenols offer unique advantages in the treatment of vitiligo. 1) Polyphenols possess potent antioxidant and anti-inflammatory properties. Vitiligo involves the damage and death of skin pigment cells, with oxidative stress being a key pathological factor. Polyphenols reduce oxidative stress and inhibit inflammatory responses by effectively scavenging free radicals and suppressing lipid peroxidation (27), which may help protect skin pigment cells. 2) Polyphenolic compounds exhibit more significant and broad activity in immune modulation. They achieve this by mechanisms including antioxidant action, regulation of cellular signaling pathways, and influencing the expression of cytokines (28, 29). For example, polyphenols can regulate the function of T cells, which play a crucial role in adaptive immune responses. While terpenoids and other natural compounds like alkaloids and glycosides also possess immunomodulatory properties, their mechanisms of action may be more specific or limited compared to polyphenols. 3) Polyphenols are abundant in sources. As crucial secondary metabolites in plants, their widespread distribution is closely tied to their roles in plant physiology, encompassing defense mechanisms (such as anti-microbial and anti-feedant properties), antioxidants, and influencing plant coloration (30). Currently, approximately 8000 types of polyphenol structures have been identified in plants (31), and their diverse biosynthetic pathways (32) make them more readily available. 4) Some polyphenols are generally considered to have lower toxicity and side effects compared to certain alkaloids and terpenoids (33). The presence of polyphenols in the natural environment and long history of human consumption indicate their safety at normal dietary intake levels.

**Figure 3:**
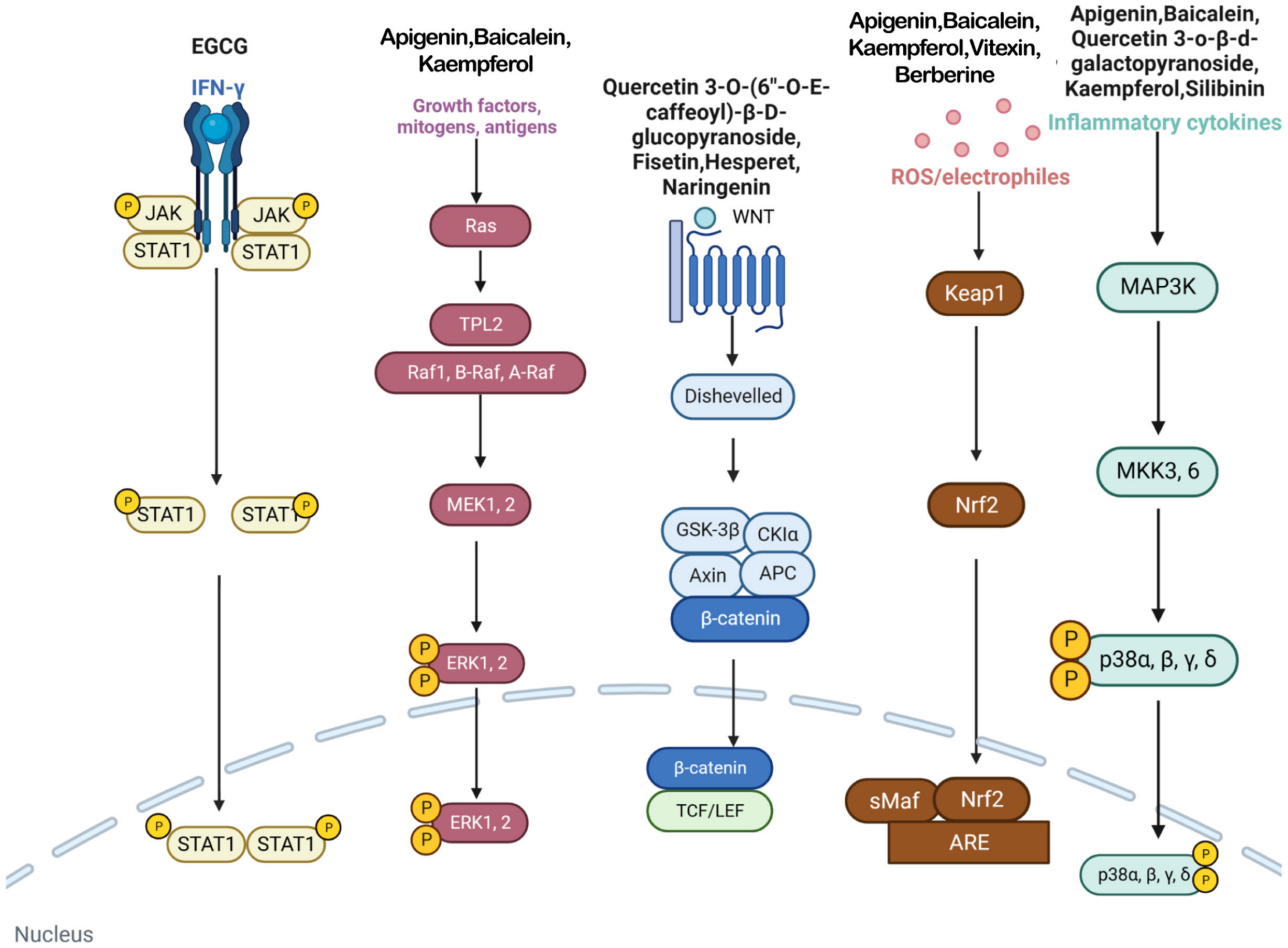
In vitiligo cells, various polyphenols target IFN-γ/JAK/STAT/CXCL10 pathway, MAPK/ERK pathway, WNT pathway, Nrf2/ARE pathway, p38 MAPK pathway and many other pathways.

This study investigates the potential role of polyphenolic compounds in treating vitiligo, a dermatological condition characterized by the loss of skin pigmentation, for which effective treatment is currently lacking. Renowned for their antioxidative and anti-inflammatory properties, polyphenolic compounds are believed to positively impact the pathogenesis of vitiligo by modulating multiple molecular targets and signaling pathways (**Figure 3**). This study systematically reviews the potential mechanisms by which polyphenolic compounds may affect the treatment of vitiligo, thereby providing a scientific basis for the development of novel therapeutic strategies and medications.

## 2 Polyphenols targeting multiple molecular targets and pathways

Polyphenols have been demonstrated to target multiple molecular pathways and modulate various cellular processes. For the treatment of vitiligo, polyphenols may aid in reducing oxidative stress, promoting melanocyte survival and regeneration, and modulating immune responses, all of which are relevant to the pathogenesis of vitiligo. Polyphenolic compounds curtail oxidative stress via diverse mechanisms. Their structure incorporates multiple phenolic hydroxyl groups (-OH) that donate hydrogen atoms or electrons, neutralizing free radicals and reducing cellular oxidative damage. Catechins in tea polyphenols, exemplified by epigallocatechin-3-gallate (EGCG), efficiently neutralize superoxide anion radicals (O_2_•−), hydroxyl radicals (•OH), and hydrogen peroxide (H_2_O_2_) (34). Furthermore, these compounds can chelate transition metals such as Fe^2+^ and Cu^2+^, thereby inhibiting their catalytic role in ROS-generating reactions. Flavonoid polyphenols chelate iron ions, preventing free radical generation catalyzed by iron (35). They also interact with cell membrane lipids to enhance stability and thwart lipid peroxidation, safeguarding cellular integrity. Quercetin, for example, integrates into lipid bilayers to increase fluidity and stability, reducing membrane vulnerability to free radicals (34). Polyphenols promote melanocyte survival and proliferation by enhancing melanocyte stem cell proliferation and differentiation, supporting pigmentation maintenance in skin and hair (36). They also regulate melanocyte function by modulating the expression of genes like the Microphthalmia-associated transcription factor (MITF), which is pivotal for melanocyte differentiation and survival, thereby promoting melanin production (37). In addition to these direct effects, polyphenols influence vitiligo pathophysiology by modulating key signaling pathways. **Table 1** summarizes the different classes of polyphenols targeting multiple molecular targets and pathways, along with their chemical structures.

**Table 1:** List of polyphenols targeting multiple molecular targets and pathways and chemical structures of polyphenols.

Classification		Compounds	Sources	Molecular targets and pathways	Effect on Melanogenesis	Refs.	Chemical structures
Flavonoids	Flavones	Apigenin	Vegetables, fruits, leaves, peas, flowers	Nrf2/ARE↑	Induction	(38)	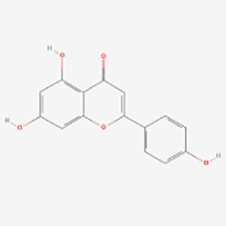
				p38 MAPK↓; JNK↓; Akt↓	Induction	(39)	
				K^+^-Cl^–^co-transport (KCC)	Induction	(40)	
		Baicalein	Scutellaria baicalensis, Rhizoma Coptidis, Rhizoma Cyperus, Eucalyptus	Nrf2/ARE↑	Induction	(41)	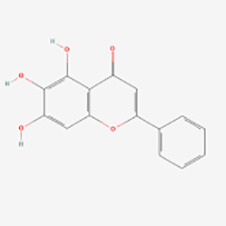
				mitochondria-dependent caspase↓; p38 MAPK↓	Induction	(42)	
		Vitexin	Zingiber officinale, hawthorn, wood bean, mung bean, bamboo	MAPK-Nrf 2/ARE↑	Induction	(43)	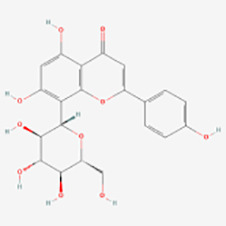
	Flavonols	Quercetin	Apples, cranberries, red onions, grapes, cherries, citrus fruits, asparagus, radishes	oxidative stress↓; ROS↓	Induction	(44)	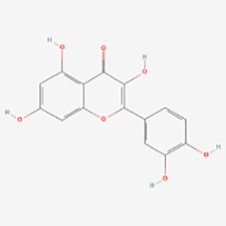
				tyrosinase↑; TRP-2↑	Induction	(45)	
				endoplasmic reticulum dilation↓; tyrosinase↑	Induction	(46)	
		Quercetin 3-*O*-β-d-galactopyranose		MITF/TYR/TYRP1/TYRP2	Induction	(47)	
				PI3K/AKT and MAPK(p38)	Induction	(48)	
		Quercetin 3-*O*-(6″-*O*-E-caffeoyl)-β-D-glucopyranoside		MAPKs and Akt/GSK3β/β-catenin	Induction	(49)	
		Kaempferol	Vegetables, fruits, herbs	oxidativestress↓; ROS↓	Induction	(44)	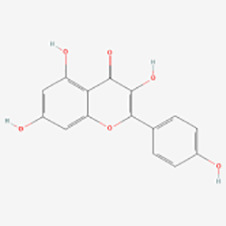
				MAPK (ERK1/2)	Induction	(50)	
				Nrf2/ARE	Induction	(51)	
				p38 MAPK/MITF	Induction	(52)	
		Silibinin	The seeds and fruits of Silybum marianum	PKA and p38 MAPK	Induction	(53)	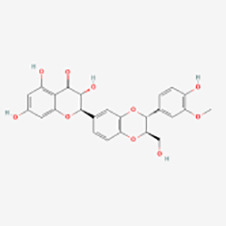
		Fisetin	Strawberries, apples, persimmons, grapes, onions, cucumbers	GSK3β/β-catenin	Induction	(54)	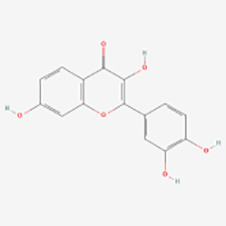
		Morin	Figs, mulberries, guavas, onions, apples	MAPK	Induction	(55)	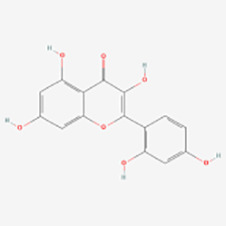
	Flavanols	EGCG	Green tea	IFN-γ/JAK/STAT/CXCL10 ↓	Induction	(56–59)	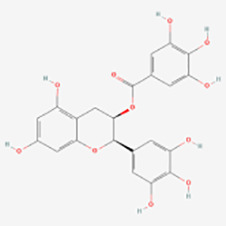
	Flavanones	Hesperetin	Citrus fruits	Wnt/β-catenin	Induction	(60)	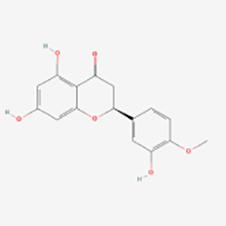
		Naringenin		Wnt/β-catenin	Induction	(61)	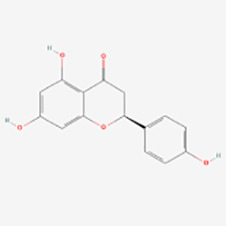
	Isoflavones	Puerarin	Pueraria mirifica	ERK1/2	Induction	(62)	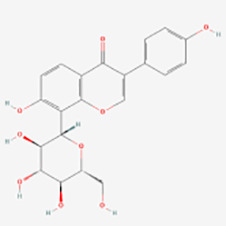
				cAMP/MITF-M	Induction	(63)	
		Icariin	Epimedium	MAPK/ERK1/2↑	Induction	(64)	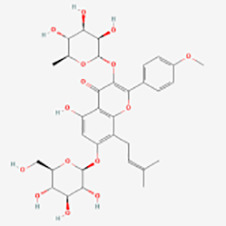
Lignans		Honokiol	Huperzine	SIRT3-OPA1↑	Induction	(65)	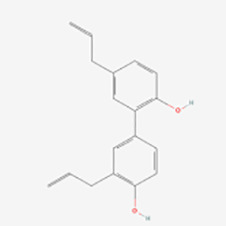
		Sesamin	Flaxseed, sesame	cAMP↑	Induction	(66)	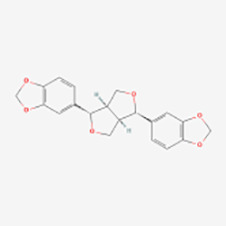
Phenolic acid		Paeonol	Paeonia suffruticosa, birch, juniper, jute plants	Nrf2/ARE↑	Induction	(67)	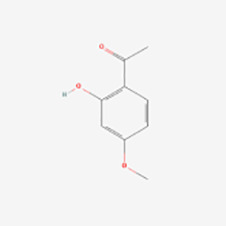
				cAMP↓	Inhibition	(68)	
Others		Capsaicin	Capsicum fruits	Smac/DIABLO, p53, NF-kB, and MAPK	Induction	(69)	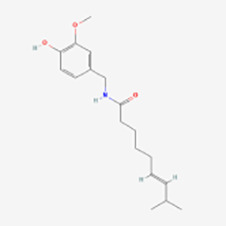
		6-Shogaol	Ginger	Nrf2/ARE	Induction	(70)	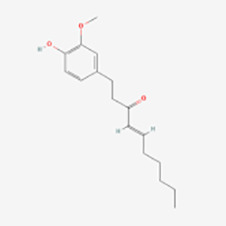
		Curcumin	Curcuma longa	p38↓; NF-kB p65↓; p53↓; Smac/DIABLO ↓	Induction	(69)	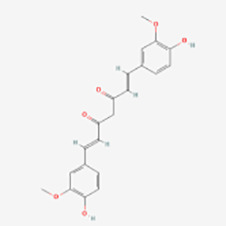

### 2.1 Flavonoids

#### 2.1.1 Flavones

##### 2.1.1.1 Apigenin

Apigenin (4′,5,7-trihydroxyflavone) is a natural flavonoid found in a wide variety of vegetables, fruits, leaves, peas, and flowers, and is the predominant type of flavonoid in celery, parsley, and chamomile (71). Apigenin glycoside, as the glycosylated form of apigenin, have been proven to possess anticancer (72–74) and antiviral (75) properties, as well as antioxidant (76), anti-inflammatory (77), and neuroprotective (78) effects. Apigenin also contributes to the alleviation of various skin injuries, including vitiligo, UV-induced skin damage, dermatitis, wounds, skin aging, and certain types of skin cancer (79). The therapeutic effect of apigenin on vitiligo may be related to enhancing antioxidant activity, activating Nrf2 pathway, inhibiting the phosphorylation of p38, JNK and Akt, and activating K^+^ - Cl^–^ cotransport (KCC).

Baoxiang Zhang et al. (38) experimentally demonstrated that apigenin-treated melanocytes showed improved viability. Apigenin also increased the expression of cellular antioxidants such as superoxide dismutase (SOD), catalase (CAT), and glutathione peroxidase (GPx), while reducing the production of the oxidative stress biomarker malondialdehyde. Additionally, apigenin treatment increased the expression and nuclear localization of the Nrf2 transcription factor. Further validation revealed that Nrf2 knockdown cells failed to exhibit significant apigenin-mediated effects on cell viability and oxidative stress, indicating the critical role of apigenin in Nrf2 modulation. The non-toxicity of apigenin and its positive effects on parameters associated with oxidative stress hold promise as a valuable therapeutic approach for the treatment of vitiligo. Mao Lin et al. (39) found that apigenin reduced the apoptosis of melanocytes induced by dopamine, a trigger of oxidative stress, by inhibiting the phosphorylation of p38, JNK and Akt, suggesting its potential as a drug candidate for vitiligo treatment. It was found that apigenin stimulated melanin synthesis in B16 mouse melanoma cells in a dose-dependent manner and regulates melanin production by activating KCC. This suggests that apigenin could promote melanogenesis by activating KCC, a membrane ion transporter in B16 cells, offering a potential therapeutic option for the treatment of vitiligo (40).

##### 2.1.1.2 Baicalein

Baicalein (5,6,7-trihydroxy-flavone) is the predominant active constituent in Scutellaria baicalensis, which is also distributed in Rhizoma Coptidis, Rhizoma Cyperus, and Eucalyptus.

Baicalein exhibits various effects, including anticancer (80, 81), improved perfusion (82), inhibition of ferroptosis (83), activation of mitochondrial autophagy (84), antimicrobial (85), and anti-inflammatory properties (86). Baicalein also enhances skin injuries such as vitiligo, skin malignancy (87), and ultraviolet damage (88). The inhibitory effect of baicalein on vitiligo may be achieved through activation of Nrf2, inhibition of oxidative stress, downregulation of the p38 MAPK pathway, and inhibition of mitochondria-dependent cystatinase activation.

Baicalein, as observed by Jingjing Ma et al. (41), effectively suppressed H_2_O_2_-induced cytotoxicity and apoptosis in human vitiligo melanocytes (PIG3V), facilitated Nrf2 nuclear translocation, and enhanced the expression of Nrf2 along with its target gene heme oxygenase-1 (HO-1). Knockdown of Nrf2 abolished the protective effect of baicalein against H_2_O_2_-induced cell injury, apoptosis and mitochondrial dysfunction. This demonstrates that baicalein boosts the antioxidant defense mechanism of PIG3V cells by activating the Nrf2 signaling pathway, providing valuable evidence for its application in the treatment of vitiligo. Bangmin Liu et al. (42) observed that baicalein exerted a notable protective effect against H_2_O_2_-induced apoptosis in human melanocytes by inhibiting mitochondria-dependent cystatinase activation and p38 MAPK pathway. Meanwhile, baicalein was able to reduce Bax/Bcl-2 ratio, cytochrome c release, cystatin-3 activation and p38 MAPK phosphorylation. These findings suggest that baicalein holds promise for treating vitiligo and can shield melanocytes from oxidative stress-induced damage via antioxidant and apoptosis inhibition pathways.

##### 2.1.1.3 Vitexin

Vitexin (apigenin-8-C-glucoside), a C-glycosylated natural flavonoid compound, is commonly present in plants including Zingiber officinale, hawthorn, wood bean, mung bean, and bamboo (89). Vitexin exhibits antioxidant (90, 91), diabetes-inhibiting (92), antibacterial (93), neuroprotective (94, 95), anti-inflammatory (96), and hepatoprotective (97) properties.

Xiao-Sha Li et al. experimentally demonstrated that vitexin inhibited H_2_O_2_-induced apoptosis of melanocytes, promoted cell proliferation, decreased intracellular reactive oxygen species levels, and suppressed the expression of inflammatory factors IL-1β and IL-17A. Additionally, oysterin activated the MAPK-Nrf2/ARE signaling pathway and induced the expression of antioxidant genes, including HO-1 and SOD. Knockdown of the Nrf2 gene reversed the protective effects of oysterin, resulting in increased expression of IL-1β, IL-17A, and ROS, and decreased expression of HO-1 and SOD. Thus, ouabain protects melanocytes from oxidative stress by activating the MAPK-Nrf2/ARE signaling pathway, providing a potential strategy for the treatment of vitiligo (43).

#### 2.1.2 Flavonols

##### 2.1.2.1 Quercetin

Quercetin (3,3′,4′,5,7-pentahydroxyflavone) is a flavonol compound present in plants with potent oxidizing properties and various biological activities (98). It is abundant in various foods including apples, cranberries, red onions, grapes, cherries, citrus fruits, asparagus, and radishes (99, 100). Quercetin has been proved to have a variety of biological activities, such as antioxidant, anti-inflammatory, anti-apoptotic, anticancer, and immunomodulatory effects (101–103).

Ziqian Xu et al. (44) observed that quercetin decreased H_2_O_2_-induced apoptosis in PIG1 cells and concurrently lowered ROS levels induced by H_2_O_2_, indicating its potential in protecting melanocytes from oxidative stress. It has been demonstrated that H_2_O_2_ induces expansion of the endoplasmic reticulum in melanocytes, inhibiting tyrosinase (TYR) output, whereas quercetin markedly mitigates these effects (46). Yun-Mi Jeong et al. (104) found that quercetin and green tea extract exhibited strong cytoprotective effects on H_2_O_2_-treated melanocytes, and various combinations of quercetin, green tea extract, and folic acid synergistically prevented cellular damage. This indicates the potential value of antioxidant combinations in vitiligo treatment and introduces a novel approach for its management. Susumu Takekoshi et al. (45) reported that quercetin promoted melanin production in the buccal follicular tissue of C3H/HeN-Jel mice by stimulating the synthesis of TYR proteins and TRP-2 proteins. With the increase of quercetin concentration, the production of melanin and the expression level of TYR protein also increased correspondingly. This suggests the potential of quercetin in treating vitiligo, though further validation through additional studies is warranted. Changhai Liu et al. (49) found that quercetin 3-O-(6″-O-E-caffeoyl)-β-D-glucopyranoside promoted melanogenesis in B16 cells. By activating the expression of MITF, TYR, tyrosinase-related protein-1 (TRP-1) and 2 (TRP-2) via the MAPKs and Akt/glycogen synthase kinase 3-beta (GSK3β)/β- catenin signaling pathway, it plays a regulatory role in melanogenesisto. Experimental findings indicate that quercetin 3-O-β-d-galactopyranoside significantly elevates protein levels of TYR, MITF, tyrosinase-associated protein 1 (TYRP 1), and tyrosinase-associated protein 2 (TYRP 2). Knockdown of MITF attenuates the effect of quercetin 3-O-β-d-galactopyranoside on melanin content, suggesting its involvement in stimulating melanogenesis through the MITF/TYR/TYRP1/TYRP2 signaling pathway. In addition, quercetin 3-O-β-d-galactopyranoside can stimulate melanogenesis and melanocyte migration, and increase melanin content by inducing the expression of MITF and its downstream target genes in human primary melanocytes. Quercetin 3-O-β-d-galactopyranoside has potential efficacy in treating vitiligo (47). Quercetin 3-O-β-d-galactopyranoside has the ability to protect human melanocytes from oxidative damage, possibly through activation of AKT, inhibition of p38 phosphorylation, and suppression of mitochondrial apoptotic signaling, which offering a novel avenue for the treatment vitiligo (48).

##### 2.1.2.2 Kaempferol

Kaempferol is a natural flavonoid present in various vegetables, fruits, and herbs, exhibiting diverse pharmacological activities such as anti-inflammatory, antioxidant, antimicrobial, anticancer, anti-apoptotic, and neuroprotective effects (105, 106).

Ziqian Xu et al. (44) observed that kaempferol reduced H_2_O_2_-induced apoptosis in PIG1 cells while concurrently decreasing ROS levels induced by H_2_O_2_. This suggests that kaempferol is able to reduce ROS in melanocytes, thereby protecting them from oxidative stress. Yihui Xie et al. (50) found that kaempferol promoted melanin synthesis in PIG1 cells by enhancing ERK1/2 phosphorylation and reduced apoptosis by inhibiting H_2_O_2_-induced ROS generation through up-regulation of HO-1 expression, as well as preventing H_2_O_2_-induced melanin reduction. Eunsun Jung et al. found that afzelin (kaempferol-3-O-alpha-L-rhamnoside) may inhibit GSK3β by activating the Nrf2/ARE signaling pathway, potentially serving as a therapeutic agent for mitigating oxidative stress-induced dermatoses (51). Research revealed afzelin upregulated the expression of MITF gene by activating the p38 MAPK pathway, subsequently promoting melanogenesis in human epidermal melanocytes (52).

##### 2.1.2.3 Silibinin

Silibinin is the main bioactive flavone extracted from the seeds and fruits of Silybum marianum (107), exhibits various biological effects such as antioxidant, anti-inflammatory, anticancer, and hepatoprotective properties (108–111).

Takuhiro Uto et al. found that silibinin significantly increased the melanin content in mouse B16-F1 and human HMV-II melanoma cells. Silibinin activated intracellular TYR activity and upregulated the expression of TYR, TRP-1, TRP-2, and MITF. Additionally, silibinin enhanced the phosphorylation of cyclic AMP response element binding protein (CREB), PKA and p38 MAPK. This proves that silibinin effectively stimulates melanogenesis by increasing the protein expression of melanogenesis enzymes via PKA and p38 pathways, leading to the phosphorylation of CREB and expression of MITF (53).

##### 2.1.2.4 Fisetin

Fisetin (3,3′,4′,7-tetrahydroxyflavone) is a naturally occurring flavonol widely distributed in fruits, vegetables and nuts, such as strawberries, apples, persimmons, grapes, onions, cucumbers and so on. It has pharmacological effects such as antioxidant, anti-inflammatory, anti-apoptosis and anticancer properties (112–116).

Menu Molagoda et al. (54) found that fisetin promoted melanogenesis by inhibiting GSK3β, activating the β-catenin signaling pathway, and upregulating MITF, TRP-1 and TRP-2. In addition, in zebrafish larval experiments, fisetin potentially promoted melanogenesis through the Wnt/β-catenin signaling pathway. These findings suggest that fisetin may be a potential herbal extract for treating vitiligo.

##### 2.1.2.5 Morin

Morin, as a dietary flavonol, widely exists in fruits and vegetables consumed daily, such as figs, mulberries, guavas, onions, apples and so on. Morin has been shown to have antioxidant, anti-inflammatory, anti-apoptotic, anticarcinogenic, and neuroprotective properties (117–121).

SeoYeon Shin et al. found that morin promotes melanogenesis by activating the ERK and p38 signaling pathways and upregulating MITF, TRP-1 and TRP-2 in B38-F16 mouse melanoma cells (55).

#### 2.1.3 Flavanols

##### 2.1.3.1 Epigallocatechin-3-gallate

EGCG is an important component of Catechins, which has been proved to possess anticancer (122), antiviral and antioxidant effects (123).

EGCG can regulate autoimmunity by reducing the number of leukocytes and CD8^+^ T cells, inhibiting the adhesion of T lymphocytes to melanocytes, suppressing inflammatory responses by downregulating TNF-α, IFN-γ, and IL-6, while simultaneously inhibiting the expression of CXCL3, CXCL5, C-FOS, CXCR4, S100B, Rab27A, TGF-Br2 genes and upregulating the expression of PI3k and EGFR genes. This process plays a role in delaying the development of vitiligo and promoting the formation of melanocytes in lesion tissues (56–58). The IFN-γ/JAK/STAT/CXCL10 pathway has been proved to be related to the occurrence and development of vitiligo, and the role of EGCG in modulating immunity and downregulating inflammatory factors may be accomplished by intervening IFN-γ/JAK/STAT/CXCL10 pathway. Weixuan Ning et al. found that EGCG directly inhibited the phosphorylation of JAK2, STAT1, and STAT3 in IFN-γ-induced human melanocytes. Additionally, the intervention of EGCG decreased the expression of CXCL10 in human melanocytes induced by IFN-γ and the protein levels of receptors such as CD11a, CXCR3, and CCR2 in T cells, ultimately inhibiting the adhesion of T cells to human melanocytes induced by IFN-γ (59). Meanwhile, downregulation of the IFN-γ/JAK/STAT/CXCL10 pathway also reduced the production of inflammatory factors such as IFN-γ.

Per-acetylated epigallocatechin-3-gallate (AcEGCG), a fully acetylated derivative of EGCG, is considered effective in protecting melanocytes from oxidative damage. Wenting Hu et al. reported that topical application of 0.5% AcEGCG cream twice daily on two patients with vitiligo resulted in controlled skin repigmentation and halted progression of pigment loss by the sixth and eighth weeks, respectively (124). In another randomized controlled trial, Wenting Hu et al. compared a group using topical EGCG to a control group treated with pimecrolimus, ultimately finding both agents effective in pigment repigmentation, with no statistically significant difference in the Vitiligo Area Scoring Index between lesions treated by the two drugs. Additionally, no severe side effects of EGCG were observed during the study (125).

#### 2.1.4 Flavanones

##### 2.1.4.1 Naringenin and hesperetin

Both naringenin and hesperidin are citrus flavanones known for their diverse biological activities, including antioxidant, anti-inflammatory, anti-mutagenic, and anticancer properties (61, 126–129).

Yu-Chun Huang et al. (61) found that naringenin enhanced melanin synthesis and TYR activity in B16-F10 cells by promoting the expression of TYR, MITF, and β-catenin, and enhancing the phosphorylation of Akt or GSK3β. This suggests that naringenin induces melanogenesis signaling through activation of the PI3K/Akt or Wnt/β-catenin pathways. It was found that hesperidin increased the phosphorylation of GSK3β by activating CREB and MAPK in the Wnt/β-catenin pathway, consequently enhancing melanogenesis (60).

#### 2.1.5 Isoflavones

##### 2.1.5.1 Puerarin

Puerarin, a major isoflavonoid extracted from Pueraria mirifica, exhibits pharmacological effects including antioxidant, anti-inflammatory, anticancer, neuroprotective, and analgesic properties (130–134).

Xiaoxia Ding et al. found that Puerarin promoted melanogenesis by inhibiting the phosphorylation and activation of the ERK1/2 signaling pathway and upregulating MITF, TRP-1 and TRP-2. Puerarin also exhibits antioxidative stress effect, which can increase the level of cAMP in cells and further promote the synthesis of melanin (62). It was found that puerarin effectively prevented vitiligo by activating the cAMP/MITF-M signaling pathway and up-regulating the expression of cAMP, MITF and its downstream pathways TYR, TRP-2 and Bcl-2, thereby stimulating melanin synthesis and increasing melanocyte survival (63).

##### 2.1.5.2 Icariin

Icariin, the primary active compound in traditional Chinese medicine Epimedium, exhibits a wide array of beneficial effects, including neuroprotection, anti-osteoporosis, anti-inflammatory, and antioxidative stress properties (135–140). Additionally, icariin shows promise in alleviating vitiligo.

Chen Hong et al. found that Epimedium extract, including icariin, could promote TYR activity, upregulate the protein expression of TYR family, thereby facilitating melanin biosynthesis via activation of the MAPK/ERK1/2 signaling pathway. In addition, Epimedium extract EFE effectively increased the number of melanosomes, accelerated melanosome maturation, and induced the transfer of melanosomes from melanocytes to keratinocytes for pigmentation via cytoplasmic transport facilitated by the growth/extension of melanocyte dendrites (64). Yan Ye et al. observed that icariin showed potent melanogenic activity, albeit without impacting the expression of regulatory factors in melanin synthesis pathways, such as TRP-1 and TRP-2. It only marginally increased the expression of TYR (140), necessitating further investigation into its specific mechanism.

### 2.2 Lignans

#### 2.2.1 Honokiol

Honokiol, as a compound with anti-inflammatory (141), anticancer (142, 143), anti-depressant (144), neuroprotective (145) properties, mainly exists in the phytomedicinal herb Huperzine. Its potential in dermatological therapy has been identified (146).

Xiuli Yi et al. (65) experimentally found that oxidative stress impairs the expression and activity of mitochondrial deacetylase sirtuin 3(SIRT3) in vitiligo melanocytes. SIRT3 deficiency promotes melanocyte apoptosis induced by oxidative stress via the SIRT3/OPA1 pathway. Honokiol significantly increased the expression of SIRT3 at mRNA and protein level, and decreased the acetylation of SOD2. This indicates that honokiol can prevent the apoptosis of vitiligo melanocytes under oxidative stress by activating the SIRT3/OPA1 pathway, offering a novel potential avenue for the treatment of vitiligo.

#### 2.2.2 Sesamin

Sesamin, a natural compound present in sesame oil and classified as a lignan, was initially isolated from flaxseed and subsequently extracted from sesame. It has gained considerable interest due to its antioxidant properties and its role in regulating blood lipid levels (147), hypertension (148), and blood glucose levels (149), among other pharmacological effects.

Zequn Jiang et al. (66) found that sesamin increased the melanin content and TYR activity in B16 melanoma cells in a dose-dependent manner. Furthermore, sesamin treatment enhanced the mRNA and protein levels of TYR. Western blot analysis showed that sesamin induced and consistently upregulated MITF. Moreover, sesamin activated CREB and PKA through a cAMP-dependent pathway. These findings demonstrate that sesamin stimulates melanogenesis in B16 cells by activating cAMP signaling and upregulating MITF and TYR.

### 2.3 Phenolic acid

#### 2.3.1 Paeonol

Paeonol, a natural compound present in the root bark of peony (Paeonia suffruticosa) and other plants, is primarily extracted from peony roots, notably in traditional Chinese medicine. Additionally, paeonol can be found in several other plant species, including certain birch, juniper, and jute plants. Studies have demonstrated that paeonol exhibits antioxidant, anti-inflammatory, antimicrobial, and antitumor activities.

Shanshan Guo et al. (67) found that salvinorin protected melanocytes from oxidative stress by activating Nrf2. They experimentally found that the intervention of paeonol can increase the cell vitality and melanogenesis of PIG1 cells treated with H_2_O_2_, and it can also relieve oxidative stress by restoring the activities of SOD, CAT and GPx. However, after knockdown of Nrf2 using siRNA, the protective effect of paeonol on antioxidant damage of PIG1 cells was eliminated. This proves that paeonol may protect melanocytes from oxidative stress by activating Nrf2, which in turn plays a role in treating vitiligo.

Interestingly, it has also been reported that paeonol can downregulate the production of melanin by reducing the phosphorylation of cAMP response element binding protein (phospho-CREB), downregulating the expression of MITF and reducing the mRNA and protein levels of TYR (150). Paeonol also inhibited melanin transfer and PAR-2 activating peptide SLIGRL-induced melanin transfer during co-culture of melanocytes and keratinocytes (68). These findings suggest that not only does paeonol fail to benefit vitiligo patients, but it may also exacerbate their symptoms. Thus, further research is necessary to elucidate the effect of paeonol on vitiligo.

### 2.4 Others

#### 2.4.1 Capsaicine

Capsaicin, a unique alkaloid primarily found in Capsicum fruits (151), has been extensively investigated for its antioxidant, anti-inflammatory, analgesic, and anticancer properties (152–155).

Matteo Becatti et al. found that capsaicin prevented apoptosis and mitochondrial damage by regulating signaling pathways in keratinocytes of vitiligo patients, including inhibiting the activation of Smac/DIABLO and NF-kB, inhibiting p38 phosphorylation and increasing ERK activity, thus improving cellular antioxidant capacity. Therefore, capsaicin shows promise in mitigating the damage of keratinocytes in vitiligo skin and halting the progression of vitiligo (69).

#### 2.4.2 6-Shogaol

6-Shogaol (6-SG) is an active compound extracted from ginger with a variety of antioxidant, anti-inflammatory, anticancer, anti-angiogenic, and neuroprotective effects (156–160).

Lingli Yang et al. (70) found that 6-Shogaol(6-SG) reduced the damage of oxidative stress to human epidermal melanocytes by activating the Nrf2/ARE pathway. In addition, 6-SG upregulated the expression of antioxidant enzymes HO-1 and quinine oxidoreductase-1, further enhancing their antioxidant effects. Therefore, 6-SG is expected to be used to prevent melanocyte loss in vitiligo.

#### 2.4.3 Curcumin

Curcumin ((E,E)-1,7- bis (4- hydroxy -3- methoxyphenyl) -1,6- heptadiene -3,5- dione), the primary active compound in the dietary spice Curcuma longa, has been utilized in Indian and traditional Chinese medicine to treat various ailments. These include pain disorders, digestive issues, menstrual challenges, skin ailments, sprains, wounds, and liver disorders (161). Due to its antioxidant properties, curcumin can potentially serve as a food preservative (162). Laboratory studies have confirmed curcumin’s antioxidant, anti-inflammatory, antiviral, antibacterial, antifungal, and anticancer properties (163–165). Recent case reports indicate that oral administration of turmeric powder (abundant in curcumin) combined with honey promotes melanin deposition and reverses vitiligo (166).

Matteo Becatti et al. (69) found that curcumin inhibited melanocyte apoptosis by increasing phosphorylation of ERK, increasing total antioxidant capacity, inhibiting intracellular ROS production and lipid peroxidation, and increasing mitochondrial activity. Matteo Becatti et al. initially observed elevated levels of activated p38, NF-kB p65 subunit, p53 and Smac/DIABLO, and low levels of phosphorylation of ERK in keratinocytes around vitiligo lesions. In contrast, intervention with curcumin significantly reduced mitochondrial superoxide production and restored mitochondrial membrane polarization compared to untreated controls. Meanwhile, curcumin decreased the expression of p38, NF-kB p65 subunit, p53 and Smac/DIABLO, increased the phosphorylation of keratinocytes around the lesion, and significantly inhibited the apoptosis of melanocytes. However, it is worth noting that curcumin has been shown to cause oxidative stress in Asian patients with acute vitiligo (167). Therefore, whether curcumin can benefit vitiligo patients still needs further clinical studies.

## 3 Conclusion and future perspectives

Vitiligo, a global autoimmune skin disorder, has a complex etiology involving factors such as autoimmunity, melanocyte dysfunction, and interactions between genetic and environmental elements. This condition not only raises significant aesthetic concerns but also carries potential psychological and social implications for the affected individuals. Therefore, continuous improvement in understanding and treating vitiligo is essential for enhancing the quality of life of patients.

Recent advancements have spotlighted the potential role of natural compounds, particularly polyphenols, in vitiligo management, owing to their unique antioxidative, anti-inflammatory, and immunomodulatory properties. These compounds are theorized to exert beneficial effects by modulating melanogenesis pathways, alleviating oxidative stress, and regulating immune responses, thus offering a complementary approach to conventional therapies. For example, the combination of curcumin cream with targeted narrow-spectrum UVB phototherapy showed slightly greater effectiveness compared to targeted narrow-spectrum UVB alone. However, there was no significant difference in the improvement of hyperpigmentation between the two groups, likely due to the small sample size (161).

Currently, research on the clinical efficacy of polyphenols in treating vitiligo remains relatively scarce, possibly due to the inherent low bioavailability of polyphenolic compounds, which is primarily determined by their chemical properties. Polyphenols are readily absorbed through the intestinal tract, but they are rapidly metabolized in the body and easily cleared by the liver, resulting in typically low plasma concentrations. Additionally, polyphenols are unstable within the body, prone to degradation in the intestinal environment, which further reduces their bioavailability. These factors limit the effective concentration of polyphenols in the body, thus affecting the efficacy and reliability of their clinical application (168). However, recent studies have demonstrated that enhancing the bioavailability of polyphenols can unlock significant potential in treating diseases like vitiligo. Various strategies to enhance the bioavailability of polyphenols have been discovered. For example, nanotechnology, which involves encapsulating polyphenols in nanocarriers to protect them from degradation in the gastrointestinal tract and enhance their absorption rates. Research indicates that nanotechnology can significantly increase their concentration and stability in the body, thereby enhancing their biological activity (169). Composite formulations can also enhance the bioavailability of polyphenols by combining them with absorption-enhancing substances such as lipids, proteins, or other enhancers. These formulations help increase their solubility and stability, thus enhancing their bioavailability. Moreover, modifying the administration route of polyphenols can increase their bioavailability. Studies have found that administering polyphenols via the skin can directly affect the skin of vitiligo patients and might be more effective than oral administration in utilizing the local therapeutic effects of polyphenols (170). With the improvement in the bioavailability of polyphenols, the potential and breadth of their clinical research are also increasing. In the future, we can anticipate more clinical trials targeting specific diseases with polyphenols, where their antioxidant and anti-inflammatory properties may be further utilized.

Despite the promising applications of polyphenolic compounds in the treatment of vitiligo, current research still has some shortcomings and problems. Due to the diversity and complexity of the structure and properties of polyphenolic compounds, the study of their pharmacological and toxicological mechanisms is more challenging. Currently, we have limited understanding of the mechanism of action of polyphenolic compounds in hypomelanosis treatment, and more in-depth studies are needed to reveal their precise molecular mechanisms. Meanwhile, the lack of systematic pharmacokinetic and pharmacodynamic studies prevents us from accurately assessing the metabolic mechanisms of polyphenolic compounds *in vivo*. Additionally, the lack of standardized methods for extracting and purifying polyphenolic compounds results in their purity and stability not being guaranteed. Moreover, the bioavailability and tissue distribution properties of polyphenolic compounds have not been clarified, which limits their use in hypomelanosis treatment. Given the safety and toxic side effects of polyphenolic compounds, their clinical efficacy needs to be verified by more large-scale clinical trials to ensure their effectiveness and safety in clinical applications. Furthermore, the mechanism of interaction between polyphenolic compounds and existing treatments is not fully understood, and further studies are needed to determine rational combination regimens.

In response to these limitations, future research should aim to address the following questions. 1) Integrative therapy and molecular mechanisms: Future research should explore the synergistic effects of combining polyphenolic compounds with established pharmacological agents, such as corticosteroids, to enhance treatment efficacy for vitiligo. This includes identifying specific polyphenols like Apigenin, Baicalein, Kaempferol, and Paeonol that may have the most promising anti-inflammatory and hypomelanotic properties, and understanding their complex molecular interactions to develop more targeted therapies. 2) Chemical structure and drug development: There is a need for a deeper understanding of how the chemical structure of polyphenolic compounds influences their biological activity. This research should aim to discover compounds with improved potency, reduced side effects, and better targeting capabilities, ensuring both the efficacy and safety of these agents in treating vitiligo. 3) Formulation science and drug delivery: The development of effective drug delivery systems is essential to enhance the bioavailability of polyphenolic compounds. This includes innovations in production, quality control, and formulation science, as well as the application of advanced technologies such as nanotechnology to protect and enhance the absorption of these compounds, thereby improving their therapeutic potential. 4) Clinical evaluation and holistic management: Future research must include comprehensive clinical evaluations to assess the efficacy and safety of polyphenolic treatments in vitiligo, including large-scale trials to determine optimal dosages, administration routes, and potential drug interactions. Furthermore, given the psychological and social impact of vitiligo, therapeutic strategies should be developed to address both the physical and mental health aspects of the condition, ensuring a holistic approach to patient care.

In summary, this review aimed at summarizing the mechanisms of natural polyphenols in the treatment of vitiligo. A clear conclusion is that these compounds have a positive effect on the treatment of vitiligo, mainly due to their antioxidative properties, immunomodulation, promotion of melanin production, and reduction of cellular apoptosis. It is crucial to further investigate the precise molecular mechanisms of these compounds in the treatment of vitiligo and explore the potential of combining them with existing therapies to enhance efficacy. We hope that this review will offer new insights for future research on utilizing natural polyphenols to target multiple pathways and targets in combating vitiligo.

## Author contributions

YY: Writing – original draft, Writing – review & editing. YD: Writing – original draft, Writing – review & editing. BC: Conceptualization, Investigation, Writing – original draft, Writing – review & editing.

## References

[1] Bergqvist C Ezzedine K. Vitiligo: A review. Dermatology. (2020) 236:571–92. doi: 10.1159/000506103 32155629

[2] Alikhan A Felsten LM Daly M Petronic-Rosic V. Vitiligo: a comprehensive overview Part I. Introduction, epidemiology, quality of life, diagnosis, differential diagnosis, associations, histopathology, etiology, and work-up. J Am Acad Dermatol. (2011) 65:473–91. doi: 10.1016/j.jaad.2010.11.061 21839315

[3] Boisseau-Garsaud AM Garsaud P Calès-Quist D Hélénon R Quénéhervé C Claire RC. Epidemiology of vitiligo in the French West Indies (Isle of Martinique). Int J Dermatol. (2000) 39:18–20. doi: 10.1046/j.1365-4362.2000.00880.x 10651958

[4] Howitz J Brodthagen H Schwartz M Thomsen K. Prevalence of vitiligo. Epidemiological survey on the Isle of Bornholm, Denmark. Arch Dermatol. (1977) 113:47–52. doi: 10.1001/archderm.113.1.47 831622

[5] Krüger C Schallreuter KU. A review of the worldwide prevalence of vitiligo in children/adolescents and adults. Int J Dermatol. (2012) 51:1206–12. doi: 10.1111/j.1365-4632.2011.05377.x 22458952

[6] Zhang Y Cai Y Shi M Jiang S Cui S Wu Y. The prevalence of vitiligo: A meta-analysis. PLoS One. (2016) 11:e0163806. doi: 10.1371/journal.pone.0163806 27673680 PMC5038943

[7] Akl J Lee S Ju HJ Parisi R Kim JY Jeon JJ. Estimating the burden of vitiligo: a systematic review and modelling study. Lancet Public Health. (2024) 9(6):e386–96. doi: 10.1016/S2468-2667(24)00026-4 38552651

[8] Rodrigues M Ezzedine K Hamzavi I Pandya AG Harris JE. New discoveries in the pathogenesis and classification of vitiligo. J Am Acad Dermatol. (2017) 77:1–13. doi: 10.1016/j.jaad.2016.10.048 28619550

[9] Ezzedine K Grimes PE Meurant JM Seneschal J Léauté-Labrèze C Ballanger F. Living with vitiligo: results from a national survey indicate differences between skin phototypes. Br J Dermatol. (2015) 173:607–9. doi: 10.1111/bjd.13839 25892476

[10] Wańkowicz-Kalińska A van den Wijngaard RM Tigges BJ Westerhof W Ogg GS Cerundolo V. Immunopolarization of CD4+ and CD8+ T cells to Type-1-like is associated with melanocyte loss in human vitiligo. Lab Invest. (2003) 83:683–95. doi: 10.1097/01.lab.0000069521.42488.1b 12746478

[11] Rashighi M Agarwal P Richmond JM Harris TH Dresser K Su MW. CXCL10 is critical for the progression and maintenance of depigmentation in a mouse model of vitiligo. Sci Transl Med. (2014) 6:223ra223. doi: 10.1126/scitranslmed.3007811 PMC4086941 24523323

[12] Damsky W King BA. JAK inhibitors in dermatology: The promise of a new drug class. J Am Acad Dermatol. (2017) 76:736–44. doi: 10.1016/j.jaad.2016.12.005 PMC6035868 28139263

[13] Jacquemin C Rambert J Guillet S Thiolat D Boukhedouni N Doutre MS. Heat shock protein 70 potentiates interferon alpha production by plasmacytoid dendritic cells: relevance for cutaneous lupus and vitiligo pathogenesis. Br J Dermatol. (2017) 177:1367–75. doi: 10.1111/bjd.15550 28380264

[14] Richmond JM Strassner JP Rashighi M Agarwal P Garg M Essien KI. Resident memory and recirculating memory T cells cooperate to maintain disease in a mouse model of vitiligo. J Invest Dermatol. (2019) 139:769–78. doi: 10.1016/j.jid.2018.10.032 PMC6431571 30423329

[15] Richmond JM Strassner JP Zapata L Jr. Garg M Riding RL Refat MA. Antibody blockade of IL-15 signaling has the potential to durably reverse vitiligo. Sci Transl Med. (2018) 10(450):eaam7710. doi: 10.1126/scitranslmed.aam7710 30021889 PMC6495055

[16] Bergqvist C Ezzedine K. Vitiligo: A focus on pathogenesis and its therapeutic implications. J Dermatol. (2021) 48:252–70. doi: 10.1111/1346-8138.15743 33404102

[17] Maresca V Roccella M Roccella F Camera E Del Porto G Passi S. Increased sensitivity to peroxidative agents as a possible pathogenic factor of melanocyte damage in vitiligo. J Invest Dermatol. (1997) 109:310–3. doi: 10.1111/1523-1747.ep12335801 9284096

[18] Bickers DR Athar M. Oxidative stress in the pathogenesis of skin disease. J Invest Dermatol. (2006) 126:2565–75. doi: 10.1038/sj.jid.5700340 17108903

[19] Frisoli ML Essien K Harris JE. Vitiligo: mechanisms of pathogenesis and treatment. Annu Rev Immunol. (2020) 38:621–48. doi: 10.1146/annurev-immunol-100919-023531 32017656

[20] Seneschal J Speeckaert R Taïeb A Wolkerstorfer A Passeron T Pandya AG. Worldwide expert recommendations for the diagnosis and management of vitiligo: Position statement from the international Vitiligo Task Force-Part 2: Specific treatment recommendations. J Eur Acad Dermatol Venereol. (2023) 37:2185–95. doi: 10.1111/jdv.19450 37715487

[21] Granci V Dupertuis YM Pichard C. Angiogenesis as a potential target of pharmaconutrients in cancer therapy. Curr Opin Clin Nutr Metab Care. (2010) 13:417–22. doi: 10.1097/MCO.0b013e3283392656 20453647

[22] Guo W Kong E Meydani M. Dietary polyphenols, inflammation, and cancer. Nutr Cancer. (2009) 61:807–10. doi: 10.1080/01635580903285098 20155620

[23] Kampa M Nifli AP Notas G Castanas E. Polyphenols and cancer cell growth. Rev Physiol Biochem Pharmacol. (2007) 159:79–113. doi: 10.1007/112_2006_0702 17551696

[24] Ramos S. Effects of dietary flavonoids on apoptotic pathways related to cancer chemoprevention. J Nutr Biochem. (2007) 18:427–42. doi: 10.1016/j.jnutbio.2006.11.004 17321735

[25] Ratz-Łyko A Arct J Majewski S Pytkowska K. Influence of polyphenols on the physiological processes in the skin. Phytother Res. (2015) 29:509–17. doi: 10.1002/ptr.5289 25586195

[26] Sajadimajd S Bahramsoltani R Iranpanah A Kumar Patra J Das G Gouda S. Advances on natural polyphenols as anticancer agents for skin cancer. Pharmacol Res. (2020) 151:104584. doi: 10.1016/j.phrs.2019.104584 31809853

[27] Gutiérrez-Del-Río I López-Ibáñez S Magadán-Corpas P Fernández-Calleja L Pérez-Valero Á Tuñón-Granda M. Terpenoids and polyphenols as natural antioxidant agents in food preservation. Antioxidants (Basel). (2021) 10(8):1264. doi: 10.3390/antiox10081264 34439512 PMC8389302

[28] Alhazmi HA Najmi A Javed SA Sultana S Al Bratty M Makeen HA. Medicinal plants and isolated molecules demonstrating immunomodulation activity as potential alternative therapies for viral diseases including COVID-19. Front Immunol. (2021) 12:637553. doi: 10.3389/fimmu.2021.637553 34054806 PMC8155592

[29] Mutha RE Tatiya AU Surana SJ. Flavonoids as natural phenolic compounds and their role in therapeutics: an overview. Futur J Pharm Sci. (2021) 7:25. doi: 10.1186/s43094-020-00161-8 33495733 PMC7816146

[30] Balasundram N Sundram K Samman S. Phenolic compounds in plants and agri-industrial by-products: Antioxidant activity, occurrence, and potential uses. Food Chem. (2006) 99:191–203. doi: 10.1016/j.foodchem.2005.07.042

[31] Tsao R. Chemistry and biochemistry of dietary polyphenols. Nutrients. (2010) 2:1231–46. doi: 10.3390/nu2121231 PMC3257627 22254006

[32] Vogt T. Phenylpropanoid biosynthesis. Mol Plant. (2010) 3:2–20. doi: 10.1093/mp/ssp106 20035037

[33] D'Archivio M Filesi C Di Benedetto R Gargiulo R Giovannini C Masella R. Polyphenols, dietary sources and bioavailability. Ann Ist Super Sanita. (2007) 43:348–61. 18209268

[34] Andrés CMC Pérez de la Lastra JM Juan CA Plou FJ Pérez-Lebeña E. Polyphenols as antioxidant/pro-oxidant compounds and donors of reducing species: relationship with human antioxidant metabolism. Processes. (2023) 11(9):2771. doi: 10.3390/pr11092771

[35] Truong VL Jeong WS. Cellular defensive mechanisms of tea polyphenols: structure-activity relationship. Int J Mol Sci. (2021) 22(17):9109. doi: 10.3390/ijms22179109 34502017 PMC8430757

[36] Mull AN Zolekar A Wang YC. Understanding melanocyte stem cells for disease modeling and regenerative medicine applications. Int J Mol Sci. (2015) 16:30458–69. doi: 10.3390/ijms161226207 PMC4691150 26703580

[37] Hughes BK Bishop CL. Current understanding of the role of senescent melanocytes in skin ageing. Biomedicines. (2022) 10(12):3111. doi: 10.3390/biomedicines10123111 36551868 PMC9775966

[38] Zhang B Wang J Zhao G Lin M Lang Y Zhang D. Apigenin protects human melanocytes against oxidative damage by activation of the Nrf2 pathway. Cell Stress Chaperones. (2020) 25:277–85. doi: 10.1007/s12192-020-01071-7 PMC7058778 31953635

[39] Lin M Lu SS Wang AX Qi XY Zhao D Wang ZH. Apigenin attenuates dopamine-induced apoptosis in melanocytes via oxidative stress-related p38, c-Jun NH2-terminal kinase and Akt signaling. J Dermatol Sci. (2011) 63:10–6. doi: 10.1016/j.jdermsci.2011.03.007 21514118

[40] Lee YS. Role of K^+^-cl^–^cotransporter in the apigenin-induced stimulation of melanogenesis in B16 melanoma cells. Yakhak Hoeji. (2008) 52:500–6.

[41] Ma J Li S Zhu L Guo S Yi X Cui T. Baicalein protects human vitiligo melanocytes from oxidative stress through activation of NF-E2-related factor2 (Nrf2) signaling pathway. Free Radic Biol Med. (2018) 129:492–503. doi: 10.1016/j.freeradbiomed.2018.10.421 30342186

[42] Liu B Jian Z Li Q Li K Wang Z Liu L. Baicalein protects human melanocytes from H_2_O_2_-induced apoptosis via inhibiting mitochondria-dependent caspase activation and the p38 MAPK pathway. Free Radic Biol Med. (2012) 53:183–93. doi: 10.1016/j.freeradbiomed.2012.04.015 22569306

[43] Li XS Tang XY Su W Li X. Vitexin protects melanocytes from oxidative stress via activating MAPK-Nrf2/ARE pathway. Immunopharmacol Immunotoxicol. (2020) 42:594–603. doi: 10.1080/08923973.2020.1835952 33045867

[44] Xu Z Xie Y Song J Huang J Shi W. Mechanism of action of a chinese herbal compound containing quercetin, luteolin, and kaempferol in the treatment of vitiligo based on network pharmacology and experimental verification. Evid Based Complement Alternat Med. (2022) 2022:7197533. doi: 10.1155/2022/7197533 36569347 PMC9788887

[45] Takekoshi S Matsuzaki K Kitatani K. Quercetin stimulates melanogenesis in hair follicle melanocyte of the mouse. Tokai J Exp Clin Med. (2013) 38:129–34. 24318284

[46] Guan C Xu W Hong W Zhou M Lin F Fu L. Quercetin attenuates the effects of H_2_O_2_ on endoplasmic reticulum morphology and tyrosinase export from the endoplasmic reticulum in melanocytes. Mol Med Rep. (2015) 11:4285–90. doi: 10.3892/mmr.2015.3242 25625855

[47] Yang B Yang Q Yan HB Yang X Lu QP. Hyperoside elevates the melanin content and promotes the migration of human melanocytes. Int J Clin Exp Med. (2017) 10(2):2953–9.

[48] Yang B Yang Q Yang X Yan HB Lu QP. Hyperoside protects human primary melanocytes against H_2_O_2_-induced oxidative damage. Mol Med Rep. (2016) 13:4613–9. doi: 10.3892/mmr.2016.5107 PMC4878558 27082158

[49] Liu C Nueraihemaiti M Zang D Edirs S Zou G Aisa HA. Quercetin 3-O-(6″-O-E-caffeoyl)-β-D-glucopyranoside, a flavonoid compound, promotes melanogenesis through the upregulation of MAPKs and akt/GSK3β/β-catenin signaling pathways. Int J Mol Sci. (2023) 24(5):4780. doi: 10.3390/ijms24054780 36902210 PMC10003212

[50] Xie Y Mei X Shi W. Kaempferol promotes melanogenesis and reduces oxidative stress in PIG1 normal human skin melanocytes. J Cell Mol Med. (2023) 27:982–90. doi: 10.1111/jcmm.17711 PMC10064034 36924030

[51] Jung E Kim JH Kim MO Lee SY Lee J. Melanocyte-protective effect of afzelin is mediated by the Nrf2-ARE signalling pathway via GSK-3β inactivation. Exp Dermatol. (2017) 26:764–70. doi: 10.1111/exd.13277 27992083

[52] Jung E Kim JH Kim MO Jang S Kang M Oh SW. Afzelin positively regulates melanogenesis through the p38 MAPK pathway. Chem Biol Interact. (2016) 254:167–72. doi: 10.1016/j.cbi.2016.06.010 27287415

[53] Uto T Ohta T Katayama K Shoyama Y. Silibinin promotes melanogenesis through the PKA and p38 MAPK signaling pathways in melanoma cells. BioMed Res. (2022) 43:31–9. doi: 10.2220/biomedres.43.31 35431290

[54] Molagoda IMN Karunarathne WAHM Park SR Choi YH Park EK Jin CY. GSK-3β-targeting fisetin promotes melanogenesis in B16F10 melanoma cells and Zebrafish larvae through β-catenin activation. Int J Mol Sci. (2020) 21(1):312. doi: 10.3390/ijms21010312 31906440 PMC6982351

[55] Shin S Ko J Kim M Song N Park K. Morin induces melanogenesis via activation of MAPK signaling pathways in B16F10 mouse melanoma cells. Molecules. (2021) 26(8):2150. doi: 10.3390/molecules26082150 33917985 PMC8068350

[56] Rashighi M Harris JE. Interfering with the IFN-γ/CXCL10 pathway to develop new targeted treatments for vitiligo. Ann Transl Med. (2015) 3:343. doi: 10.3978/j.issn.2305-5839.2015.11.36 26734651 PMC4690998

[57] Singh BN Shankar S Srivastava RK. Green tea catechin, epigallocatechin-3-gallate (EGCG): mechanisms, perspectives and clinical applications. Biochem Pharmacol. (2011) 82:1807–21. doi: 10.1016/j.bcp.2011.07.093 PMC4082721 21827739

[58] Zhu Y Wang S Lin F Li Q Xu A. The therapeutic effects of EGCG on vitiligo. Fitoterapia. (2014) 99:243–51. doi: 10.1016/j.fitote.2014.08.007 25128425

[59] Ning W Wang S Dong X Liu D Fu L Jin R. Epigallocatechin-3-gallate (EGCG) suppresses the trafficking of lymphocytes to epidermal melanocytes via inhibition of JAK2: its implication for vitiligo treatment. Biol Pharm Bull. (2015) 38:1700–6. doi: 10.1248/bpb.b15-00331 26345342

[60] Huang YC Liu KC Chiou YL. Melanogenesis of murine melanoma cells induced by hesperetin, a Citrus hydrolysate-derived flavonoid. Food Chem Toxicol. (2012) 50:653–9. doi: 10.1016/j.fct.2012.01.012 22266363

[61] Huang YC Yang CH Chiou YL. Citrus flavanone naringenin enhances melanogenesis through the activation of Wnt/β-catenin signalling in mouse melanoma cells. Phytomedicine. (2011) 18:1244–9. doi: 10.1016/j.phymed.2011.06.028 21802267

[62] Ding X Mei E Hu M Zhou C Li X Cai L. Effect of puerarin on melanogenesis in human melanocytes and vitiligo mouse models and the underlying mechanism. Phytother Res. (2019) 33:205–13. doi: 10.1002/ptr.6218 30421463

[63] Park WS Kwon O Yoon TJ Chung JH. Anti-graying effect of the extract of Pueraria thunbergiana via upregulation of cAMP/MITF-M signaling pathway. J Dermatol Sci. (2014) 75:153–5. doi: 10.1016/j.jdermsci.2014.05.003 24924521

[64] Hong C Yang L Zhang Y Li Y Wu H. Epimedium brevicornum Maxim. Extract exhibits pigmentation by melanin biosynthesis and melanosome biogenesis/transfer. Front Pharmacol. (2022) 13:963160. doi: 10.3389/fphar.2022.963160 36249817 PMC9557186

[65] Yi X Guo W Shi Q Yang Y Zhang W Chen X. SIRT3-dependent mitochondrial dynamics remodeling contributes to oxidative stress-induced melanocyte degeneration in vitiligo. Theranostics. (2019) 9:1614–33. doi: 10.7150/thno.30398 PMC6485185 31037127

[66] Jiang Z Li S Liu Y Deng P Huang J He G. Sesamin induces melanogenesis by microphthalmia-associated transcription factor and tyrosinase up-regulation via cAMP signaling pathway. Acta Biochim Biophys Sin (Shanghai). (2011) 43:763–70. doi: 10.1093/abbs/gmr078 21896570

[67] Guo S Zhang Q. Paeonol protects melanocytes against hydrogen peroxide-induced oxidative stress through activation of Nrf2 signaling pathway. Drug Dev Res. (2021) 82:861–9. doi: 10.1002/ddr.21793 33491230

[68] Xie SH Chen ZQ Ma PC. Down-regulation of melanin synthesis and transfer by paeonol and its mechanisms. Am J Chin Med. (2007) 35:139–51. doi: 10.1142/S0192415X07004692 17265558

[69] Becatti M Prignano F Fiorillo C Pescitelli L Nassi P Lotti T. The involvement of Smac/DIABLO, p53, NF-kB, and MAPK pathways in apoptosis of keratinocytes from perilesional vitiligo skin: Protective effects of curcumin and capsaicin. Antioxid Redox Signal. (2010) 13:1309–21. doi: 10.1089/ars.2009.2779 20085492

[70] Yang L Yang F Teng L Katayama I. 6-Shogaol Protects Human Melanocytes against Oxidative Stress through Activation of the Nrf2-Antioxidant Response Element Signaling Pathway. Int J Mol Sci. (2020) 21(10):3537. doi: 10.3390/ijms21103537 32429495 PMC7279012

[71] Tang D Chen K Huang L Li J. Pharmacokinetic properties and drug interactions of apigenin, a natural flavone. Expert Opin Drug Metab Toxicol. (2017) 13:323–30. doi: 10.1080/17425255.2017.1251903 27766890

[72] Jiang ZB Wang WJ Xu C Xie YJ Wang XR Zhang YZ. Luteolin and its derivative apigenin suppress the inducible PD-L1 expression to improve anti-tumor immunity in KRAS-mutant lung cancer. Cancer Lett. (2021) 515:36–48. doi: 10.1016/j.canlet.2021.05.019 34052328

[73] Wang M Firrman J Liu L Yam K. A review on flavonoid apigenin: dietary intake, ADME, antimicrobial effects, and interactions with human gut microbiota. BioMed Res Int. (2019) 2019:7010467. doi: 10.1155/2019/7010467 31737673 PMC6817918

[74] Xu L Zhang Y Tian K Chen X Zhang R Mu X. Apigenin suppresses PD-L1 expression in melanoma and host dendritic cells to elicit synergistic therapeutic effects. J Exp Clin Cancer Res. (2018) 37:261. doi: 10.1186/s13046-018-0929-6 30373602 PMC6206930

[75] Saadh MJ Jaber SA Alaraj M Alafnan A. Apigenin inhibits infectious bronchitis virus replication in ovo. Eur Rev Med Pharmacol Sci. (2022) 26:5367–71. doi: 10.26355/eurrev_202208_29403 35993630

[76] Kashyap P Shikha D Thakur M Aneja A. Functionality of apigenin as a potent antioxidant with emphasis on bioavailability, metabolism, action mechanism and in *vitro* and in *vivo* studies: A review. J Food Biochem. (2022) 46:e13950. doi: 10.1111/jfbc.13950 34569073

[77] Rahimi A Alimohammadi M Faramarzi F Alizadeh-Navaei R Rafiei A. The effects of apigenin administration on the inhibition of inflammatory responses and oxidative stress in the lung injury models: a systematic review and meta-analysis of preclinical evidence. Inflammopharmacology. (2022) 30:1259–76. doi: 10.1007/s10787-022-00994-0 35661071

[78] Nabavi SF Khan H D'Onofrio G Šamec D Shirooie S Dehpour AR. Apigenin as neuroprotective agent: Of mice and men. Pharmacol Res. (2018) 128:359–65. doi: 10.1016/j.phrs.2017.10.008 29055745

[79] Majma Sanaye P Mojaveri MR Ahmadian R Sabet Jahromi M Bahramsoltani R. Apigenin and its dermatological applications: A comprehensive review. Phytochemistry. (2022) 203:113390. doi: 10.1016/j.phytochem.2022.113390 35998830

[80] Yan W Ma X Zhao X Zhang S. Baicalein induces apoptosis and autophagy of breast cancer cells via inhibiting PI3K/AKT pathway in *vivo* and vitro. Drug Des Devel Ther. (2018) 12:3961–72. doi: 10.2147/DDDT.S181939 PMC6248272 30510404

[81] Yu M Qi B Xiaoxiang W Xu J Liu X. Baicalein increases cisplatin sensitivity of A549 lung adenocarcinoma cells via PI3K/Akt/NF-κB pathway. BioMed Pharmacother. (2017) 90:677–85. doi: 10.1016/j.biopha.2017.04.001 28415048

[82] Li M Meng Z Yu S Li J Wang Y Yang W. Baicalein ameliorates cerebral ischemia-reperfusion injury by inhibiting ferroptosis via regulating GPX4/ACSL4/ACSL3 axis. Chem Biol Interact. (2022) 366:110137. doi: 10.1016/j.cbi.2022.110137 36055377

[83] Wan Y Shen K Yu H Fan W. Baicalein limits osteoarthritis development by inhibiting chondrocyte ferroptosis. Free Radic Biol Med. (2023) 196:108–20. doi: 10.1016/j.freeradbiomed.2023.01.006 36657732

[84] Ke PY Chang CW Hsiao YC. Baicalein activates Parkin-dependent mitophagy through NDP52 and OPTN. Cells. (2022) 11(7):1132. doi: 10.3390/cells11071132 35406696 PMC8997844

[85] Wang Y Su J Zhou Z Yang J Liu W Zhang Y. Baicalein resensitizes multidrug-resistant gram-negative pathogens to doxycycline. Microbiol Spectr. (2023) 11:e0470222. doi: 10.1128/spectrum.04702-22 37070985 PMC10269726

[86] Dinda B Dinda M Dinda S De UC. An overview of anti-SARS-CoV-2 and anti-inflammatory potential of baicalein and its metabolite baicalin: Insights into molecular mechanisms. Eur J Med Chem. (2023) 258:115629. doi: 10.1016/j.ejmech.2023.115629 37437351

[87] Ma GZ Liu CH Wei B Qiao J Lu T Wei HC. Baicalein inhibits DMBA/TPA-induced skin tumorigenesis in mice by modulating proliferation, apoptosis, and inflammation. Inflammation. (2013) 36:457–67. doi: 10.1007/s10753-012-9566-y 23108957

[88] Kimura Y Sumiyoshi M. Effects of baicalein and wogonin isolated from Scutellaria baicalensis roots on skin damage in acute UVB-irradiated hairless mice. Eur J Pharmacol. (2011) 661:124–32. doi: 10.1016/j.ejphar.2011.04.033 21549115

[89] He M Min JW Kong WL He XH Li JX Peng BW. A review on the pharmacological effects of vitexin and isovitexin. Fitoterapia. (2016) 115:74–85. doi: 10.1016/j.fitote.2016.09.011 27693342

[90] Babaei F Moafizad A Darvishvand Z Mirzababaei M Hosseinzadeh H Nassiri-Asl M. Review of the effects of vitexin in oxidative stress-related diseases. Food Sci Nutr. (2020) 8:2569–80. doi: 10.1002/fsn3.1567 PMC7300089 32566174

[91] Zhang Y Wang D Yang L Zhou D Zhang J. Purification and characterization of flavonoids from the leaves of Zanthoxylum bungeanum and correlation between their structure and antioxidant activity. PloS One. (2014) 9:e105725. doi: 10.1371/journal.pone.0105725 25157400 PMC4144902

[92] Abdulai IL Kwofie SK Gbewonyo WS Boison D Puplampu JB Adinortey MB. Multitargeted effects of vitexin and isovitexin on diabetes mellitus and its complications. ScientificWorldJournal. (2021) 2021:6641128. doi: 10.1155/2021/6641128 33935599 PMC8055414

[93] Das MC Samaddar S Jawed JJ Ghosh C Acharjee S Sandhu P. Vitexin alters Staphylococcus aureus surface hydrophobicity to obstruct biofilm formation. Microbiol Res. (2022) 263:127126. doi: 10.1016/j.micres.2022.127126 35914415

[94] Lima LKF Pereira SKS Junior RDSS Santos FPDS Nascimento AS Feitosa CM. A brief review on the neuroprotective mechanisms of vitexin. BioMed Res Int. (2018) 2018:4785089. doi: 10.1155/2018/4785089 30627560 PMC6304565

[95] Yahaya MAF Zolkiffly SZI Moklas MAM Hamid HA Stanslas J Zainol M. Possible epigenetic role of vitexin in regulating neuroinflammation in Alzheimer's disease. J Immunol Res. (2020) 2020:9469210. doi: 10.1155/2020/9469210 32258178 PMC7085883

[96] Zhao CR Yang FF Cui Q Wang D Zhou Y Li YS. Vitexin inhibits APEX1 to counteract the flow-induced endothelial inflammation. Proc Natl Acad Sci USA. (2021) 118(48):e2115158118. doi: 10.1073/pnas.2115158118 34810252 PMC8640790

[97] Zhang C Li S Sun C Liu L Fang Y Yang X. Vitexin ameliorates glycochenodeoxycholate-induced hepatocyte injury through SIRT6 and JAK2/STAT3 pathways. Iran J Basic Med Sci. (2021) 24:1717–25. doi: 10.22038/ijbms.2021.59424.13196 PMC8976905 35432812

[98] Qi W Qi W Xiong D Long M. Quercetin: its antioxidant mechanism, antibacterial properties and potential application in prevention and control of toxipathy. Molecules. (2022) 27(19):6545. doi: 10.3390/molecules27196545 36235082 PMC9571766

[99] Hosseini A Razavi BM Banach M Hosseinzadeh H. Quercetin and metabolic syndrome: A review. Phytother Res. (2021) 35:5352–64. doi: 10.1002/ptr.7144 34101925

[100] Shen P Lin W Deng X Ba X Han L Chen Z. Potential implications of quercetin in autoimmune diseases. Front Immunol. (2021) 12:689044. doi: 10.3389/fimmu.2021.689044 34248976 PMC8260830

[101] Cui Z Zhao X Amevor FK Du X Wang Y Li D. Therapeutic application of quercetin in aging-related diseases: SIRT1 as a potential mechanism. Front Immunol. (2022) 13:943321. doi: 10.3389/fimmu.2022.943321 35935939 PMC9355713

[102] Murakami A Ashida H Terao J. Multitargeted cancer prevention by quercetin. Cancer Lett. (2008) 269:315–25. doi: 10.1016/j.canlet.2008.03.046 18467024

[103] Shabir I Kumar Pandey V Shams R Dar AH Dash KK Khan SA. Promising bioactive properties of quercetin for potential food applications and health benefits: A review. Front Nutr. (2022) 9:999752. doi: 10.3389/fnut.2022.999752 36532555 PMC9748429

[104] Jeong YM Choi YG Kim DS Park SH Yoon JA Kwon SB. Cytoprotective effect of green tea extract and quercetin against hydrogen peroxide-induced oxidative stress. Arch Pharm Res. (2005) 28:1251–6. doi: 10.1007/BF02978208 16350851

[105] Dong X Zhou S Nao J. Kaempferol as a therapeutic agent in Alzheimer's disease: Evidence from preclinical studies. Ageing Res Rev. (2023) 87:101910. doi: 10.1016/j.arr.2023.101910 36924572

[106] Jantas D Malarz J Le TN Stojakowska A. Neuroprotective Properties of Kempferol Derivatives from Maesa membranacea against Oxidative Stress-Induced Cell Damage: An Association with Cathepsin D Inhibition and PI3K/Akt Activation. Int J Mol Sci. (2021) 22(19):10363. doi: 10.3390/ijms221910363 34638702 PMC8509010

[107] Crocenzi FA Roma MG. Silymarin as a new hepatoprotective agent in experimental cholestasis: new possibilities for an ancient medication. Curr Med Chem. (2006) 13:1055–74. doi: 10.2174/092986706776360950 16611084

[108] Abenavoli L Capasso R Milic N Capasso F. Milk thistle in liver diseases: past, present, future. Phytother Res. (2010) 24:1423–32. doi: 10.1002/ptr.3207 20564545

[109] Federico A Dallio M Loguercio C. Silymarin/silybin and chronic liver disease: A marriage of many years. Molecules. (2017) 22(2):191. doi: 10.3390/molecules22020191 28125040 PMC6155865

[110] Romanucci V Pagano R Lembo A Capasso D Di Gaetano S Zarrelli A. Phosphodiester silybin dimers powerful radical scavengers: A antiproliferative activity on different cancer cell lines. Molecules. (2022) 27(5):1702. doi: 10.3390/molecules27051702 35268803 PMC8911775

[111] Sun R Xu D Wei Q Zhang B Aa J Wang G. Silybin ameliorates hepatic lipid accumulation and modulates global metabolism in an NAFLD mouse model. BioMed Pharmacother. (2020) 123:109721. doi: 10.1016/j.biopha.2019.109721 31865143

[112] Hou DX Fukuda M Johnson JA Miyamori K Ushikai M Fujii M. Fisetin induces transcription of NADPH:quinone oxidoreductase gene through an antioxidant responsive element-involved activation. Int J Oncol. (2001) 18:1175–9. doi: 10.3892/ijo.18.6.1175 11351248

[113] Khan N Asim M Afaq F Abu Zaid M Mukhtar H. A novel dietary flavonoid fisetin inhibits androgen receptor signaling and tumor growth in athymic nude mice. Cancer Res. (2008) 68:8555–63. doi: 10.1158/0008-5472.CAN-08-0240 PMC2954499 18922931

[114] Khan N Syed DN Ahmad N Mukhtar H. Fisetin: a dietary antioxidant for health promotion. Antioxid Redox Signal. (2013) 19:151–62. doi: 10.1089/ars.2012.4901 PMC3689181 23121441

[115] Kim SC Kang SH Jeong SJ Kim SH Ko HS Kim SH. Inhibition of c-Jun N-terminal kinase and nuclear factor κ B pathways mediates fisetin-exerted anti-inflammatory activity in lipopolysccharide-treated RAW264. 7 Cells Immunopharmacol Immunotoxicol. (2012) 34:645–50. doi: 10.3109/08923973.2011.648270 22239491

[116] Sung B Pandey MK Aggarwal BB. Fisetin, an inhibitor of cyclin-dependent kinase 6, down-regulates nuclear factor-kappaB-regulated cell proliferation, antiapoptotic and metastatic gene products through the suppression of TAK-1 and receptor-interacting protein-regulated IkappaBalpha kinase activation. Mol Pharmacol. (2007) 71:1703–14. doi: 10.1124/mol.107.034512 17387141

[117] Ishola IO Awogbindin IO Olubodun-Obadun TG Oluwafemi OA Onuelu JE Adeyemi OO. Morin ameliorates rotenone-induced Parkinson disease in mice through antioxidation and anti-neuroinflammation: gut-brain axis involvement. Brain Res. (2022) 1789:147958. doi: 10.1016/j.brainres.2022.147958 35654119

[118] Khamchai S Chumboatong W Hata J Tocharus C Suksamrarn A Tocharus J. Morin protects the blood-brain barrier integrity against cerebral ischemia reperfusion through anti-inflammatory actions in rats. Sci Rep. (2020) 10:13379. doi: 10.1038/s41598-020-70214-8 32770144 PMC7414849

[119] Lee KM Lee Y Chun HJ Kim AH Kim JY Lee JY. Neuroprotective and anti-inflammatory effects of morin in a murine model of Parkinson's disease. J Neurosci Res. (2016) 94:865–78. doi: 10.1002/jnr.23764 27265894

[120] Mottaghi S Abbaszadeh H. The anticarcinogenic and anticancer effects of the dietary flavonoid, morin: Current status, challenges, and future perspectives. Phytother Res. (2021) 35:6843–61. doi: 10.1002/ptr.7270 34498311

[121] Nowak E Sypniewski D Bednarek I. Morin exerts anti-metastatic, anti-proliferative and anti-adhesive effect in ovarian cancer cells: an in *vitro* studies. Mol Biol Rep. (2020) 47:1965–78. doi: 10.1007/s11033-020-05293-x 32020427

[122] Lecumberri E Dupertuis YM Miralbell R Pichard C. Green tea polyphenol epigallocatechin-3-gallate (EGCG) as adjuvant in cancer therapy. Clin Nutr. (2013) 32:894–903. doi: 10.1016/j.clnu.2013.03.008 23582951

[123] Lambert JD Elias RJ. The antioxidant and pro-oxidant activities of green tea polyphenols: a role in cancer prevention. Arch Biochem Biophys. (2010) 501:65–72. doi: 10.1016/j.abb.2010.06.013 20558130 PMC2946098

[124] Hu W Jin R Lin F Lei J Ma Y Xu AE. Repigmentation in two patients with vitiligo on AcEGCG 0.5% cream. Clin Exp Dermatol. (2022) 47:1760–1. doi: 10.1111/ced.15211 35731108

[125] Hu W Zhang L Lin F Lei J Ma Y Xu AE. Topical epigallocatechin-3-gallate in the treatment of vitiligo. Australas J Dermatol. (2021) 62:e404–7. doi: 10.1111/ajd.13612 34046892

[126] Francis AR Shetty TK Bhattacharya RK. Modulating effect of plant flavonoids on the mutagenicity of N-methyl-N'-nitro-N-nitrosoguanidine. Carcinogenesis. (1989) 10:1953–5. doi: 10.1093/carcin/10.10.1953 2676226

[127] Li C Schluesener H. Health-promoting effects of the citrus flavanone hesperidin. Crit Rev Food Sci Nutr. (2017) 57:613–31. doi: 10.1080/10408398.2014.906382 25675136

[128] Raso GM Meli R Di Carlo G Pacilio M Di Carlo R. Inhibition of inducible nitric oxide synthase and cyclooxygenase-2 expression by flavonoids in macrophage J774A.1. Life Sci. (2001) 68:921–31. doi: 10.1016/S0024-3205(00)00999-1 11213362

[129] So FV Guthrie N Chambers AF Carroll KK. Inhibition of proliferation of estrogen receptor-positive MCF-7 human breast cancer cells by flavonoids in the presence and absence of excess estrogen. Cancer Lett. (1997) 112:127–33. doi: 10.1016/S0304-3835(96)04557-0 9066718

[130] Chiu PY Leung HY Leong PK Chen N Zhou L Zuo Z. Danshen-Gegen decoction protects against hypoxia/reoxygenation-induced apoptosis by inhibiting mitochondrial permeability transition via the redox-sensitive ERK/Nrf2 and PKCϵ/mKATP pathways in H9c2 cardiomyocytes. Phytomedicine. (2012) 19:99–110. doi: 10.1016/j.phymed.2011.07.002 21899994

[131] Fang X Zhang Y Cao Y Shan M Song D Ye C. Studies on chemical composition of pueraria lobata and its anti-tumor mechanism. Molecules. (2022) 27(21):7253. doi: 10.3390/molecules27217253 36364084 PMC9657109

[132] Han J Yu CQ Shen W. [Inhibitory effects of puerarin on invasion and metastasis of oophoroma cells HO-8910]. Zhongguo Zhong Xi Yi Jie He Za Zhi. (2009) 29:632–5. 19852298

[133] Hongyun H Tao G Pengyue Z Liqiang Y Yihao D. Puerarin provides a neuroprotection against transient cerebral ischemia by attenuating autophagy at the ischemic penumbra in neurons but not in astrocytes. Neurosci Lett. (2017) 643:45–51. doi: 10.1016/j.neulet.2017.02.009 28192195

[134] Xu C Xu W Xu H Xiong W Gao Y Li G. Role of puerarin in the signalling of neuropathic pain mediated by P2X3 receptor of dorsal root ganglion neurons. Brain Res Bull. (2012) 87:37–43. doi: 10.1016/j.brainresbull.2011.10.007 22044944

[135] Guo J Li F Wu Q Gong Q Lu Y Shi J. Protective effects of icariin on brain dysfunction induced by lipopolysaccharide in rats. Phytomedicine. (2010) 17:950–5. doi: 10.1016/j.phymed.2010.03.007 20382007

[136] Li C Li Q Mei Q Lu T. Pharmacological effects and pharmacokinetic properties of icariin, the major bioactive component in Herba Epimedii. Life Sci. (2015) 126:57–68. doi: 10.1016/j.lfs.2015.01.006 25634110

[137] Nian H Ma MH Nian SS Xu LL. Antiosteoporotic activity of icariin in ovariectomized rats. Phytomedicine. (2009) 16:320–6. doi: 10.1016/j.phymed.2008.12.006 19269147

[138] Sha D Li L Ye L Liu R Xu Y. Icariin inhibits neurotoxicity of beta-amyloid by upregulating cocaine-regulated and amphetamine-regulated transcripts. Neuroreport. (2009) 20:1564–7. doi: 10.1097/WNR.0b013e328332d345 19858766

[139] Tao F Qian C Guo W Luo Q Xu Q Sun Y. Inhibition of Th1/Th17 responses via suppression of STAT1 and STAT3 activation contributes to the amelioration of murine experimental colitis by a natural flavonoid glucoside icariin. Biochem Pharmacol. (2013) 85:798–807. doi: 10.1016/j.bcp.2012.12.002 23261528

[140] Ye Y Chou GX Wang H Chu JH Yu ZL. Flavonoids, apigenin and icariin exert potent melanogenic activities in murine B16 melanoma cells. Phytomedicine. (2010) 18:32–5. doi: 10.1016/j.phymed.2010.06.004 20638260

[141] Lee YJ Lee YM Lee CK Jung JK Han SB Hong JT. Therapeutic applications of compounds in the Magnolia family. Pharmacol Ther. (2011) 130:157–76. doi: 10.1016/j.pharmthera.2011.01.010 21277893

[142] Prasher P Fatima R Sharma M Tynybekov B Alshahrani AM Ateşşahin DA. Honokiol and its analogues as anticancer compounds: Current mechanistic insights and structure-activity relationship. Chem Biol Interact. (2023) 386:110747. doi: 10.1016/j.cbi.2023.110747 37816447

[143] Rauf A Patel S Imran M Maalik A Arshad MU Saeed F. Honokiol: An anticancer lignan. BioMed Pharmacother. (2018) 107:555–62. doi: 10.1016/j.biopha.2018.08.054 30114639

[144] Zhang B Wang PP Hu KL Li LN Yu X Lu Y. Antidepressant-like effect and mechanism of action of honokiol on the mouse lipopolysaccharide (LPS) depression model. Molecules. (2019) 24(11):2035. doi: 10.3390/molecules24112035 31141940 PMC6600641

[145] Wang D Cao L Zhou X Wang G Ma Y Hao X. Mitigation of honokiol on fluoride-induced mitochondrial oxidative stress, mitochondrial dysfunction, and cognitive deficits through activating AMPK/PGC-1α/Sirt3. J Hazard Mater. (2022) 437:129381. doi: 10.1016/j.jhazmat.2022.129381 35752048

[146] Li Y Liang C Zhou X. The application prospects of honokiol in dermatology. Dermatol Ther. (2022) 35:e15658. doi: 10.1111/dth.15658 35726011 PMC9541939

[147] Zhang R Yu Y Hu S Zhang J Yang H Han B. Sesamin ameliorates hepatic steatosis and inflammation in rats on a high-fat diet via LXRα and PPARα. Nutr Res. (2016) 36:1022–30. doi: 10.1016/j.nutres.2016.06.015 27632923

[148] Kuo TN Lin CS Li GD Kuo CY Kao SH. Sesamin inhibits cervical cancer cell proliferation by promoting p53/PTEN-mediated apoptosis. Int J Med Sci. (2020) 17:2292–8. doi: 10.7150/ijms.48955 PMC7484641 32922194

[149] Huang SM Chuang CH Rejano CJF Tayo LL Hsieh CY Huang SK. Sesamin: A promising therapeutic agent for ameliorating symptoms of diabetes. Molecules. (2023) 28(21):7255. doi: 10.3390/molecules28217255 37959677 PMC10649669

[150] Bu J Ma PC Chen ZQ Zhou WQ Fu YJ Li LJ. Inhibition of MITF and tyrosinase by paeonol-stimulated JNK/SAPK to reduction of phosphorylated CREB. Am J Chin Med. (2008) 36:245–63. doi: 10.1142/S0192415X08005758 18457359

[151] Reyes-Escogido Mde L Gonzalez-Mondragon EG Vazquez-Tzompantzi E. Chemical and pharmacological aspects of capsaicin. Molecules. (2011) 16:1253–70. doi: 10.3390/molecules16021253 PMC6259610 21278678

[152] Brederson JD Kym PR Szallasi A. Targeting TRP channels for pain relief. Eur J Pharmacol. (2013) 716:61–76. doi: 10.1016/j.ejphar.2013.03.003 23500195

[153] Clark R Lee SH. Anticancer properties of capsaicin against human cancer. Anticancer Res. (2016) 36:837–43. 26976969

[154] Galano A Martínez A. Capsaicin, a tasty free radical scavenger: mechanism of action and kinetics. J Phys Chem B. (2012) 116:1200–8. doi: 10.1021/jp211172f 22188587

[155] Kim CS Kawada T Kim BS Han IS Choe SY Kurata T. Capsaicin exhibits anti-inflammatory property by inhibiting IkB-a degradation in LPS-stimulated peritoneal macrophages. Cell Signal. (2003) 15:299–306. doi: 10.1016/S0898-6568(02)00086-4 12531428

[156] Bischoff-Kont I Primke T Niebergall LS Zech T Fürst R. Ginger constituent 6-shogaol inhibits inflammation- and angiogenesis-related cell functions in primary human endothelial cells. Front Pharmacol. (2022) 13:844767. doi: 10.3389/fphar.2022.844767 35281937 PMC8914105

[157] da Rosa N de Medeiros FD de Oliveira J Laurentino AOM Peretti EM Machado RS. 6-Shogaol improves behavior and memory in Wistar rats prenatally exposed to lipopolysaccharide. Int J Dev Neurosci. (2022) 82:39–49. doi: 10.1002/jdn.10157 34755374

[158] da Rosa N de Medeiros FD de Oliveira J Laurentino AOM Peretti EM Machado RS. 6-shogaol exerts a neuroprotective factor in offspring after maternal immune activation in rats. Dev Neurosci. (2022) 44:13–22. doi: 10.1159/000519992 34695825

[159] Dugasani S Pichika MR Nadarajah VD Balijepalli MK Tandra S Korlakunta JN. Comparative antioxidant and anti-inflammatory effects of [6]-gingerol, [8]-gingerol, [10]-gingerol and [6]-shogaol. J Ethnopharmacol. (2010) 127:515–20. doi: 10.1016/j.jep.2009.10.004 19833188

[160] Pei XD He ZL Yao HL Xiao JS Li L Gu JZ. 6-Shogaol from ginger shows anti-tumor effect in cervical carcinoma via PI3K/Akt/mTOR pathway. Eur J Nutr. (2021) 60:2781–93. doi: 10.1007/s00394-020-02440-9 33416981

[161] Anand P Kunnumakkara AB Newman RA Aggarwal BB. Bioavailability of curcumin: problems and promises. Mol Pharm. (2007) 4:807–18. doi: 10.1021/mp700113r 17999464

[162] Thangapazham RL Sharma A Maheshwari RK. Beneficial role of curcumin in skin diseases. Adv Exp Med Biol. (2007) 595:343–57. doi: 10.1007/978-0-387-46401-5_15 17569219

[163] Kuttan R Sudheeran PC Josph CD. Turmeric and curcumin as topical agents in cancer therapy. Tumori. (1987) 73:29–31. doi: 10.1177/030089168707300105 2435036

[164] Maheshwari RK Singh AK Gaddipati J Srimal RC. Multiple biological activities of curcumin: a short review. Life Sci. (2006) 78:2081–7. doi: 10.1016/j.lfs.2005.12.007 16413584

[165] Thangapazham RL Sharma A Maheshwari RK. Multiple molecular targets in cancer chemoprevention by curcumin. AAPS J. (2006) 8:E443–449. doi: 10.1208/aapsj080352 PMC2761050 17025261

[166] Alshaikh AA Bharti RK. Spontaneous reversal of vitiligo, a rare phenomenon reported in a case in Saudi Arabia with an insight into metabolic biochemical derangements. Medicina (Kaunas). (2023) 59(3):427. doi: 10.3390/medicina59030427 36984427 PMC10053937

[167] Schallreuter KU Rokos H. Turmeric (curcumin): a widely used curry ingredient, can contribute to oxidative stress in Asian patients with acute vitiligo. Indian J Dermatol Venereol Leprol. (2006) 72:57–9. doi: 10.4103/0378-6323.19722 16481714

[168] Manach C Scalbert A Morand C Rémésy C Jiménez L. Polyphenols: food sources and bioavailability. Am J Clin Nutr. (2004) 79:727–47. doi: 10.1093/ajcn/79.5.727 15113710

[169] Teng H Chen L. Polyphenols and bioavailability: an update. Crit Rev Food Sci Nutr. (2019) 59:2040–51. doi: 10.1080/10408398.2018.1437023 29405736

[170] Johnson JJ Nihal M Siddiqui IA Scarlett CO Bailey HH Mukhtar H. Enhancing the bioavailability of resveratrol by combining it with piperine. Mol Nutr Food Res. (2011) 55:1169–76. doi: 10.1002/mnfr.201100117 PMC3295233 21714124

